# Intranasal bovine/human parainfluenza virus 3 vaccine candidates expressing human metapneumovirus wild-type or pre-fusion F protein elicit protective immunity against human metapneumovirus in hamsters

**DOI:** 10.1128/jvi.01145-25

**Published:** 2025-11-25

**Authors:** Sirle Saul, Bibha Dahal, Cindy Luongo, Xueqiao Liu, Lijuan Yang, Celia Santos, Jin Dai, Linong Zhang, Ursula J. Buchholz, Shirin Munir

**Affiliations:** 1 RNA Viruses Section, Laboratory of Infectious Diseases, National Institute of Allergy and Infectious Diseases, National Institutes of Health 35037, Bethesda, Maryland, USA; 2 Sanofi, Waltham, Massachusetts, USA; Emory University School of Medicine, Atlanta, Georgia, USA

**Keywords:** human parainfluenza virus 3, human metapneumovirus, live-attenuated vaccine, intranasal vaccine, mucosal vaccine, prefusion F

## Abstract

Human metapneumovirus (HMPV) and human parainfluenza virus 3 (HPIV3) are among the leading causes of severe pediatric respiratory illness worldwide, and there are no licensed vaccines. Here, we describe live-attenuated pediatric intranasal vaccine candidates for dual protection against HMPV and HPIV3. An attenuated chimeric bovine/HPIV3 (B/HPIV3) based on bovine PIV3, with fusion (F) and hemagglutinin-neuraminidase surface glycoproteins replaced by those of HPIV3, was designed to express HMPV subgroup A F protein, the major protective HMPV antigen. Eight viruses were generated, each containing a different version of the HMPV F ORF, including wild-type (wt), codon-optimized (BBopt or GSopt) to increase protein expression, a chimeric form (GSopt-TMCT) with the transmembrane/cytoplasmic tail (TMCT) sequence replaced with that of BPIV3 F to increase HMPV F packaging, or versions with amino acid substitutions (BBopt-D185P, GSopt-D185P, BBopt-N46V/T160F, and GSopt-N46V/T160F) to improve stability in pre-fusion form and enhance immunogenicity. In hamsters, a single intranasal dose conferred serum HMPV- and HPIV3-neutralizing antibodies and provided protection in the upper and lower respiratory tract against HMPV challenge, similar to that afforded by wt HMPV infection. Control hamsters immunized intramuscularly with two doses of HMPV pre-fusion subunit antigens stabilized by D185P or N46V/T160F mutations developed HMPV-neutralizing antibody titers, similar to those induced by the B/HPIV3/HMPV-F candidates, and were similarly protected against HMPV challenge in the lungs. However, in the upper respiratory tract, B/HPIV3/HMPV-F candidates were significantly more protective than the subunit antigens. Thus, B/HPIV3 expressing HMPV wt or pre-fusion F are promising pediatric vaccine candidates for intranasal immunization against HMPV and HPIV3.

**IMPORTANCE:**

Human metapneumovirus (HMPV) and human parainfluenza virus 3 (HPIV3) are important pediatric pathogens and need effective vaccines. We developed live-attenuated chimeric bovine/HPIV3 (B/HPIV3) expressing the fusion (F) protein of HMPV subgroup A as a bivalent pediatric intranasal vaccine against HPIV3 and HMPV. To enhance HMPV F immunogenicity, three approaches were evaluated: codon optimization to increase expression, structure-based mutations to stabilize HMPV F in pre-fusion conformation, or replacement of the HMPV F transmembrane/cytoplasmic domains with those of BPIV3 F to increase incorporation in the virus particle. In hamsters, a single intranasal dose of any of the B/HPIV3s expressing HMPV F was as immunogenic and protective against HMPV as a prior HMPV infection. In the upper respiratory tract, these provided greater protection against HMPV replication than the two intramuscular doses of the adjuvanted HMPV pre-F subunit antigens. B/HPIV3 expressing a codon-optimized pre-fusion stabilized HMPV F was advanced to a pediatric clinical study.

## INTRODUCTION

Human parainfluenza virus 3 (HPIV3) and human metapneumovirus (HMPV) are second to respiratory syncytial virus (RSV) as the most common causes of acute lower respiratory illness in children under 5 years of age (1). Currently, there are neither approved vaccines to protect against HPIV3 or HMPV illness nor are there effective antiviral treatments.

HPIVs are associated with a significant global pediatric health burden, especially in the developing world, and account for 4% of the childhood mortality due to respiratory disease (1). Primary HPIV3 infection occurs early in life, with approximately 60%–80% of children experiencing their first infection by 2 years of age (2, 3). HPIV3 is an enveloped, single-stranded, negative-sense RNA virus classified in the family *Paramyxoviridae*, genus *Respirovirus*, and encodes six structural proteins (N, P, M, F, HN, and L). The virus enters the host cell via the coordinated functions of the two envelope glycoproteins, the fusion (F) and hemagglutinin-neuraminidase (HN). F and HN are the virus neutralization and major protective antigens and are the targets for vaccine development (4–6).

HMPV, first described in 2001 (7), causes acute respiratory illness in people of all ages, and virtually every child is infected with HMPV by the age of 5 years. Globally, HMPV is estimated to be responsible for 11% of acute lower respiratory illness (ALRI) among young children (8). Based on worldwide estimates for 2018, 14.2 million ALRI cases, including about 500,000 hospital admissions and 11,300 deaths in children younger than 5 years of age, were attributed to HMPV (9). HMPV is an enveloped, single-stranded RNA virus in the family *Pneumoviridae*, genus *Metapneumovirus* and has two genetic lineages (A and B), each with two sublineages (A1, A2, B1, and B2) (10, 11). The non-segmented negative-sense RNA genome contains eight genes in the order 3′-N-P-M-F-M2-SH-G-L-5′ that encode nine proteins, including three envelope glycoproteins, the small hydrophobic (SH), attachment (G), and fusion (F) glycoproteins. The HMPV F protein, which mediates fusion of the viral envelope with the host cell membrane during viral entry, is the major neutralization and protective antigen of HMPV. Moreover, the F proteins of lineages A and B are highly conserved, displaying at least 95% identity at the amino acid level, and are closely related antigenically (10–14). Therefore, the HMPV F protein is a key target for vaccine development.

HMPV F is a class I glycoprotein, synthesized as a fusion-inactive precursor F_0_, and requires proteolytic processing by trypsin-like proteases or transmembrane serine proteases to become fusion competent (7, 15). Cleavage of F_0_ exposes the fusion peptide and generates F_2_ and F_1_ subunits that remain covalently linked by disulfide bonds. This mature fusogenic form of the HMPV F protein is incorporated into the virion as a trimer in a metastable pre-fusion (pre-F) conformation which, during the fusion/entry process, undergoes structural changes into a highly stable post-fusion (post-F) conformation (16). During propagation of HMPV in cell culture, addition of trypsin is required to mediate efficient cleavage of the F_0_ protein.

In general, the pre-F form of viral fusion proteins is more immunogenic than the post-F form, which is particularly true for the closely related respiratory syncytial virus (RSV) F protein (17, 18). In the case of RSV F, unique and highly protective epitopes are present only in the pre-F conformation. The most potent RSV-neutralizing antibodies recognize these pre-F-specific sites and account for most of the RSV-neutralizing activity in human sera (17). For HMPV, initial studies with pre- and post-F proteins showed that both forms effectively deplete HMPV-neutralizing antibodies from human sera, demonstrating that both possess neutralizing epitopes (19). Consistently, a soluble pre-F stabilized version D185P and the non-stabilized version were both immunogenic and protective in experimental animals when administered intramuscularly (IM) as subunit vaccines (19). Additional pre-F stabilizing mutations in the HMPV F protein were recently described (20–23), including the N46V/T160F substitutions, which provide intraprotomer interface stabilization and greater thermostability than D185P. Site-specific monoclonal antibody binding suggested that N46V/T160F retains all the pre-F specific antigenic sites, including sites Ø and V (23). Intramuscular immunization with HMPV F-N46V/T160F subunit antigen induced significantly higher serum HMPV-neutralizing antibody titers compared with post-F antigen in mice and therefore seemed to be a superior vaccine antigen (20–23). Most studies comparing the immunogenicity of HMPV pre- and post-F forms have been conducted using soluble F subunit antigen, administered as adjuvanted vaccines in at least two intramuscular (IM) doses (20–23).

The immunogenicity and protective properties of the HMPV stabilized pre-F forms, when expressed by a replicating live virus and delivered intranasally, have not been evaluated. HPIV3 and HMPV infect young children via the respiratory route, and attack rates are high during the first year of life. Intranasal immunization early in life with a bivalent single-dose live vaccine would be advantageous. Here, we designed a bivalent intranasal vaccine to induce both mucosal and systemic immunity against HPIV3 and HMPV. We selected recombinant bovine/human parainfluenza virus 3 (B/HPIV3) to express different versions of HMPV F from an added gene. B/HPIV3 is a chimeric virus that consists of bovine PIV3 (BPIV3) (Kansas strain) in which the F and HN genes have been replaced with those from HPIV3 (JS strain) (24). B/HPIV3 was previously developed as a live vaccine candidate against HPIV3 and was well-tolerated and immunogenic in young children (25). Moreover, B/HPIV3 expressing the RSV F protein, designed as a bivalent HPIV3/RSV vaccine candidate, was well-tolerated in children >2 months of age (26) (Clinicaltrials.gov NCT00686075). In the present study, eight versions of HMPV F (subgroup A2) were designed and expressed by B/HPIV3 to evaluate parameters that might improve immunogenicity. We characterized these vaccine candidates *in vitro*, followed by evaluation as intranasal vaccines in the hamster model. For comparison, we also included the soluble HMPV F antigen containing the N46V/T160F pre-F stabilizing substitutions (23), and the first-generation pre-F subunit containing the D185P substitution (19), both adjuvanted with alum-85 and administered via the intramuscular (IM) route. Based on the results obtained with HMPV F subunit antigens (23), N46V/T160F was a top candidate for expression by B/HPIV3 and virus rescued in Vero cells, a suitable substrate for GMP vaccine manufacture, was further characterized as a potential lead candidate.

## RESULTS

### Generation of recombinant B/HPIV3 expressing HMPV F protein

**Fig 1:**
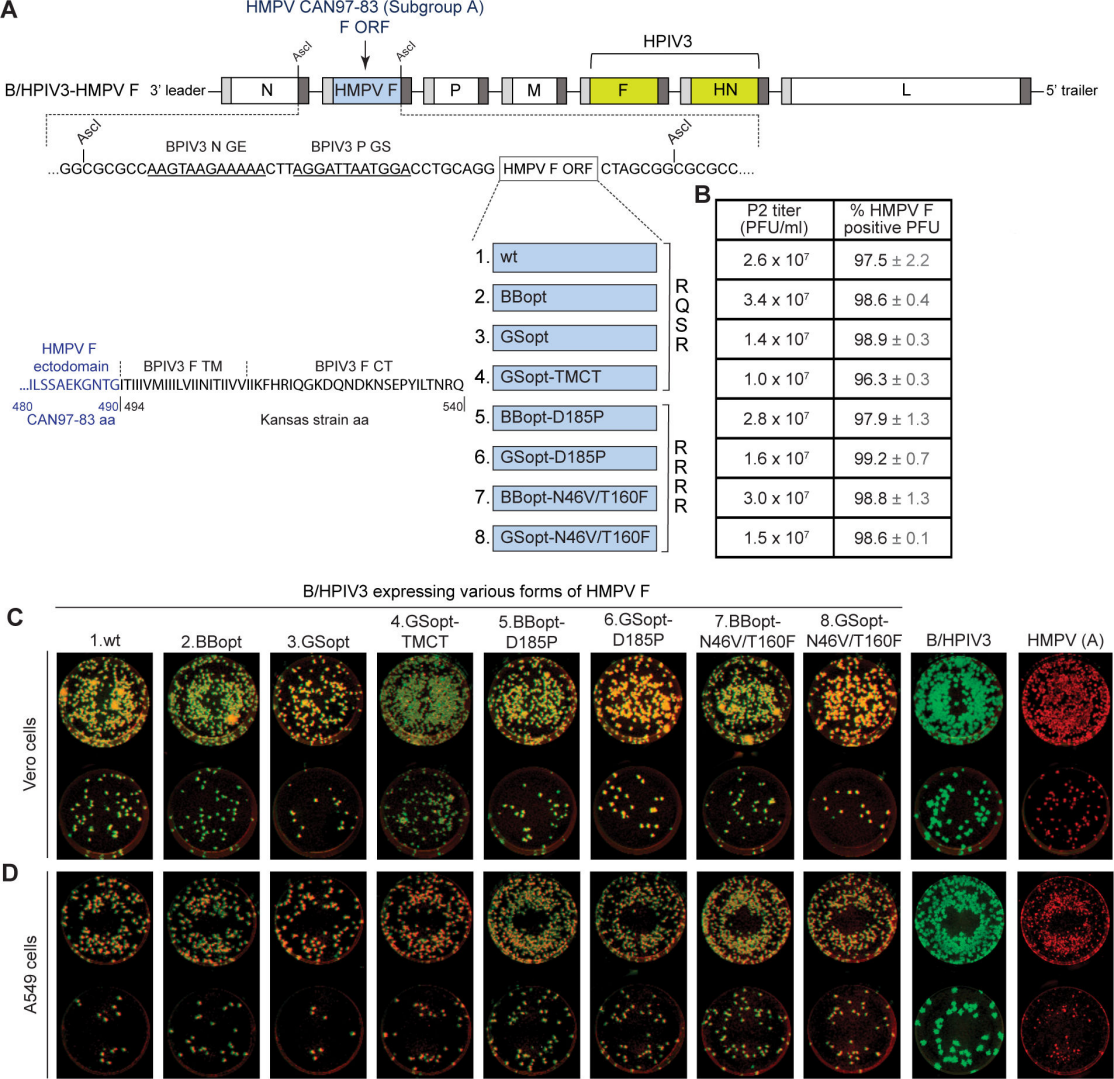
Genome design and *in vitro* stability of B/HPIV3 expressing HMPV F protein. (**A**) Schematic overview of the genomic map of the recombinant viruses. The B/HPIV3 backbone contains BPIV3 genes N, P, M, and L (white) and HPIV3 genes F and HN (green). The gene start (GS) and gene end (GE) transcription signals are shown as light and dark gray boxes, respectively, flanking each ORF. HMPV (CAN97-83) F wt ORF and its seven modified derivatives (blue) (described in Materials and Methods and numbered 1–8) were inserted at an *Asc*I site between the N and P genes yielding eight virus constructs. The enlargement shows the DNA sequence of the flanking regions containing an *Asc*I site, followed by a BPIV3 gene junction containing (in 5′ to 3′ order) the sequence for the N gene-end signal (AAGTAAGAAAAA), intergenic sequence (CTT), and a gene-start signal (AGGATTAATGGA) directing transcription of the additional gene, followed by a short noncoding region, the respective HMPV F ORF, followed by a second *Asc*I site. The second enlargement shows the amino acid sequence for GSopt-TMCT, including the downstream end of the HMPV F ectodomain (HMPV CAN97-83 aa 480-490) linked to the transmembrane (TM) and cytoplasmic tail (CT) of BPIV3 F (strain Kansas, aa 494-540). (**B–D**) Passage 2 (P2) viral stock titers and the stability of HMPV F analyzed by dual-antigen immuno-plaque assay. Titers of LLC-MK2-cell grown P2 virus stocks and percent PFU positive for HMPV F expression (±SD) following titration in Vero cells (**B**). Vero (**C**) and A549 (**D**) cells in 24-well plates were infected with serially diluted P2 virus stocks, covered with a methylcellulose overlay, and incubated for 7 days. Cells were fixed, and plaques were immunostained for the PIV3 and HMPV F antigens. Plaques appear yellow on detection of both HMPV F and PIV3 antigens or green on detection of PIV3 antigens only. Plaques from two serial dilution steps are shown for each virus.

To develop a bivalent HPIV3/HMPV vaccine, we used B/HPIV3 as a vector to express the HMPV F protein from an added gene. We designed eight different versions of the HMPV F coding sequence, derived from strain CAN97-83, subgroup A (Fig. 1A), to identify the most immunogenic vaccine candidate. Construct one contains the wild-type (wt) HMPV F ORF of HMPV strain CAN97-83. Constructs 2 and 3 also encode the wt HMPV F protein, but the HMPV F ORFs were codon-optimized either by Biobasic (BBopt; construct 2) or Genscript (GSopt; construct 3) to increase protein synthesis. These versions serve to compare the effects of codon optimization on HMPV F protein expression. To make construct 4 (GSopt-TMCT), the transmembrane and cytoplasmic tail (TMCT) domains (nt 1,474–1,620) of the GSopt HMPV F ORF (construct 3) were replaced with those of BPIV3 F to potentially enhance packaging into the B/HPIV3 particle. This was based on previous studies evaluating B/HPIV3 expressing the RSV F protein from an added gene; we found that the TMCT substitution significantly enhanced the packaging of RSV F in the B/HPIV3 virion and increased the immunogenicity of RSV F (27). Thus, constructs 3 and 4 serve to compare the effects of TMCT modification on vaccine virus attenuation, immunogenicity, and efficacy.

We included the previously described pre-F stabilized HMPV F proteins containing either D185P (19) (constructs 5 and 6) or N46V/T160F (constructs 7 and 8) amino acid substitutions that have been shown to enhance pre-F stability and immunogenicity of an HMPV subunit vaccine in mice (23). Additionally, in constructs 5 to 8, we replaced the naturally occurring trypsin-dependent HMPV F cleavage sequence (RQSR, aa 99–102) with RRRR, creating a furin cleavage motif that is naturally present in the F protein of a closely related type A avian metapneumovirus, allowing cleavage by furin-like proteases (28). Based on our previous findings that insertion at the second gene position of B/HPIV3 allows for efficient and stable expression of the heterologous gene with minimal impact on B/HPIV3 replication (29), each HMPV F gene was inserted between the N and *P* genes of the B/HPIV3 cDNA (Fig. 1A).

### Rescue and genetic stability of B/HPIV3 expressing HMPV F protein

All eight viruses were readily rescued from T7 polymerase-driven plasmids (Materials and Methods) by transfection of BHK21 cells, clone BSR-T7/5, stably expressing T7 RNA polymerase (passage 1 [P1]). Viruses were amplified in rhesus monkey kidney epithelial (LLC-MK2) cells to obtain passage 2 (P2) stocks that were used for all subsequent experiments. In parallel, GSopt-N46V/T160F (23) (construct 8) was also rescued in Vero cells, a suitable substrate for GMP vaccine manufacture, with T7 RNA polymerase expressed by a co-transfected expression plasmid. This was followed by amplification of a P2 stock (B/HPIV3/GSopt-N46V/T160F#) in Vero cells, simulating the recovery and amplification of the clinical study material. Sequencing of the full-length viral genomes of the P2 stocks did not identify any adventitious mutations in any of the viruses. We determined viral titers, the stability of HMPV F protein expression, and plaque phenotype by a fluorescent dual-antigen staining plaque assay on Vero and human lung epithelial A549 cells. During immunostaining, the PIV3 and HMPV F antigens were pseudo-colored green and red, respectively, so that the plaques formed by B/HPIV3 expressing HMPV F would appear yellow, and those that lost HMPV F expression would appear green. All recovered viruses replicated efficiently, and their P2 stocks reached final titers (Fig. 1B) comparable to that of the B/HPIV3 backbone. Dual staining for PIV3 and HMPV F antigens (yellow or orange) was observed for 96%–99% of the plaques in Vero cells (Fig. 1B and C), indicating that the HMPV F inserts were well tolerated and were stably expressed. The one exception was GSopt-TMCT (construct 4), for which stocks from initial recoveries contained substantial proportions of PFU that had lost HMPV F expression. Following additional recoveries, a P2 stock was identified in which 96% of the plaques were positive for HMPV F (Fig. 1B); nonetheless, these observations revealed that this insert was somewhat unstable.

Plaques were generally homogeneous in size for each virus but appeared slightly larger in Vero (Fig. 1C) than in A549 (Fig. 1D) cells. In both A549 and Vero cells, plaques of all B/HPIV3 vectors expressing HMPV F were much smaller than those of the B/HPIV3 control without insert, suggesting restricted replication, possibly because of a larger genome size due to the insert. Furthermore, the virus expressing GSopt-TMCT developed smaller plaques compared to other viruses (Fig. 1C and D, construct 4), suggesting that the TMCT substitution imposes an additional restrictive effect on virus growth. In Vero cells, a stronger HMPV F staining intensity was seen for GSopt viruses (except GSopt-TMCT), implying a higher level of HMPV F expression, likely because of codon optimization (Fig. 1C; Fig. S1A), but this difference was not evident in A549 cells (Fig. 1D; Fig. S1B).

### Virus replication and stability of HMPV F expression in cell culture

**Fig 2:**
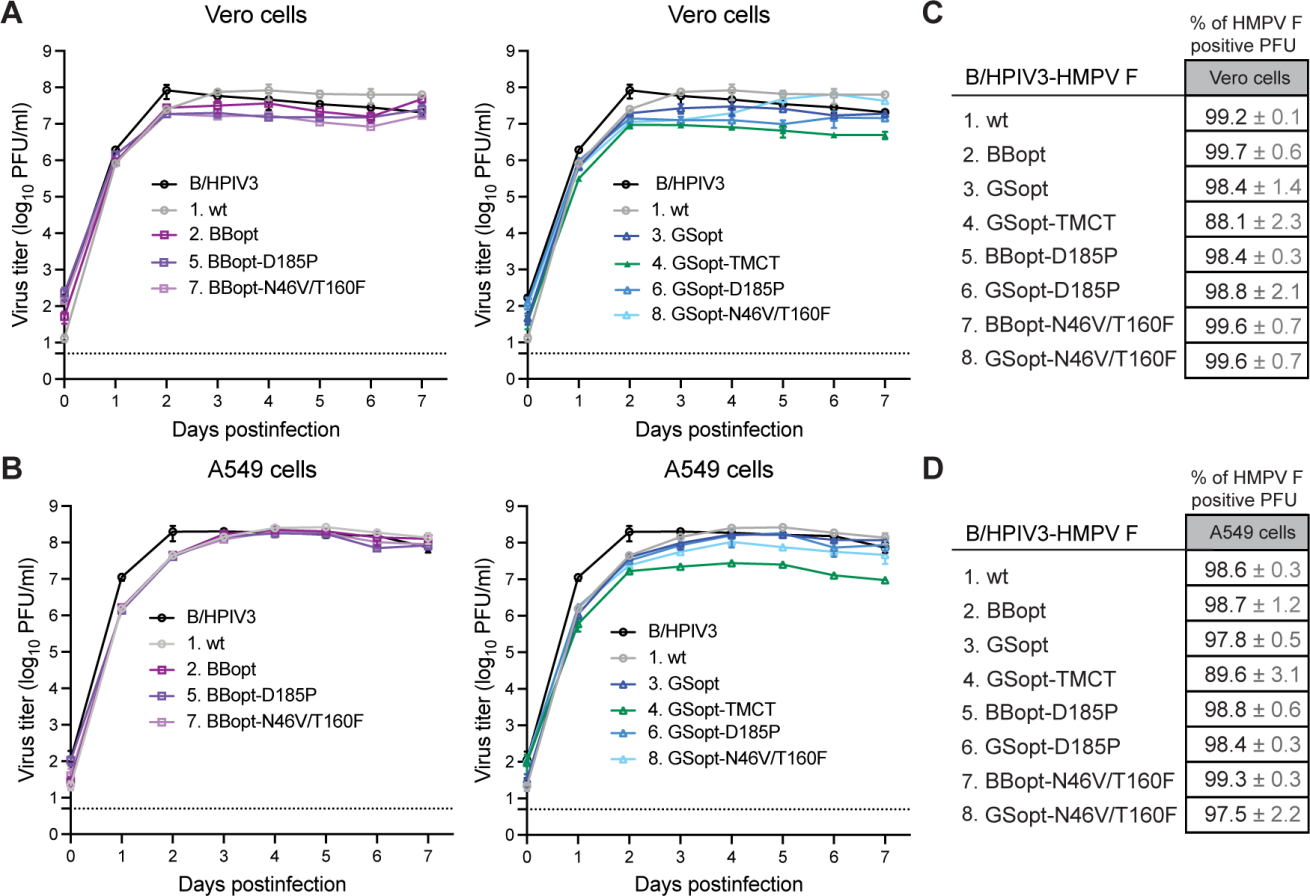
Replication kinetics of recombinant viruses. (**A, B**) Multicycle growth curves of B/HPIV3 vectors on Vero and A549 cells. Vero (**A**) and A549 (**B**) cells in six-well plates were infected in triplicate with indicated viruses at an MOI of 0.1 PFU per cell and incubated at 32°C for a total of 7 days. At 24-h intervals, aliquots of culture medium were collected and replaced with equal volume of fresh medium. Virus titers were determined by immunoplaque titration on Vero cells. The mean titers (±SD) are shown as two separate graphs for visual clarity. (**C, D**) Percentage of HMPV F-positive plaque-forming units (PFU) in the day 7 samples from Vero (**C**) and A549 (**D**) cells.

Multicycle replication experiments were performed in Vero and A549 cells, starting at an input multiplicity of infection (MOI) of 0.1 PFU per cell. Samples were collected at indicated time points, and viral titers and the stability of HMPV F expression were evaluated by HPIV3/HMPV F dual-staining immunoplaque assay (Fig. 2). The results revealed a slight delay in the accumulation of infectious progeny for B/HPIV3 expressing HMPV F compared with B/HPIV3 without the insert in Vero and A549 cells (Fig. 2A and B). B/HPIV3 control, without insert, reached its peak titer on day 2 post-infection (p.i.), while B/HPIV3 expressing HMPV wt F reached the same peak titers on days 4–5 p.i.. Relative to B/HPIV3 expressing HMPV wt F, the viruses expressing modified versions of HMPV F protein had similar growth kinetics. Only the replication of B/HPIV3 expressing GSopt-TMCT was significantly restricted, showing approximately 10-fold lower peak titers compared with B/HPIV3 or B/HPIV3 containing HMPV wt F in both cell lines. After multiple rounds of replication, staining for both PIV3 and HMPV F antigens was observed for 97%–99% of plaques on day 7 p.i. in both cell lines (Fig. 2C and D), with the exception of GSopt-TMCT, for which 88% and 89% of plaques expressed HMPV F in Vero and A549 cells, respectively, indicating a decline in the stability of HMPV F expression compared with its P2 stock. Similar growth kinetics were observed in A549 cells using a lower input MOI of 0.01 PFU per cell (Fig. S2). By the end of the growth curve experiment in Vero cells, more cell death, indicated by a higher number of floating cells, was observed for viruses expressing GSopt pre-F (viruses 6 and 8) compared with their BBopt counterparts (viruses 5 and 7) (data not shown). This could be due to a higher expression of HMPV F protein from the GSopt ORF, as observed by plaque immunostaining in Vero cells (Fig. S1). These differences were less obvious in A549 cells.

### Viral protein expression in Vero and A549 cells

**Fig 3:**
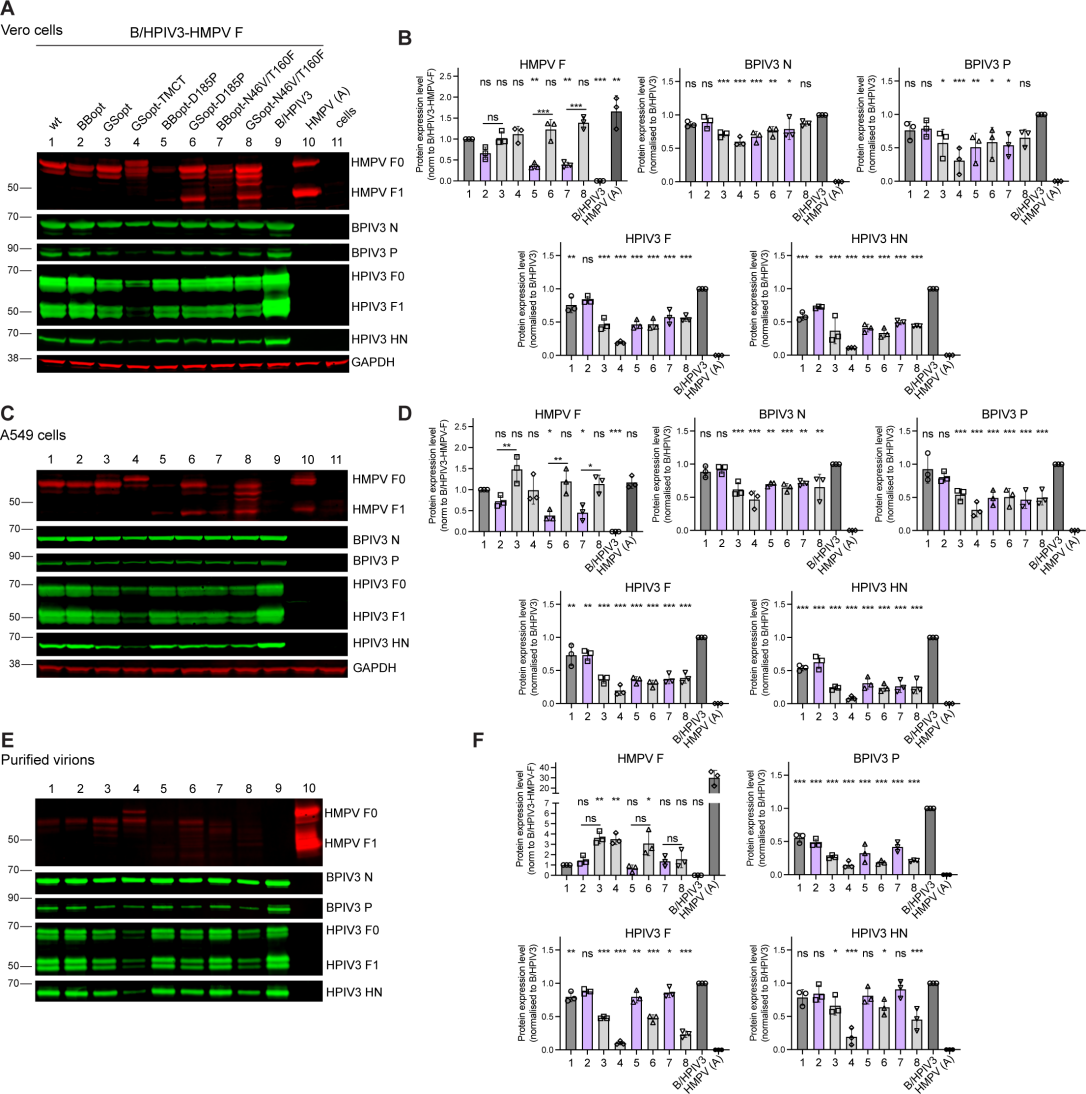
Viral proteins in the infected cell lysates and purified virions. (**A–D**), Vero (**A, B**) and A549 (**C, D**) cells in six-well plates were infected with indicated viruses or mock-infected at an MOI of 3 PFU/cell and incubated at 32°C for 48 h. Cell lysates were prepared and analyzed by Western blotting. BPIV3 N and *P* (apparent molecular weight ~60 and 75 kDa, respectively), HPIV3 F (F_0_: ∼70 kDa; F_1_: ∼48 kDa) and HN (~65 kDa), and HMPV F (~60 and ~47 kDa for F_0_ and F_1_, respectively) were detected using specific primary antibodies (Materials and Methods) followed by infrared dye-conjugated secondary antibodies. GAPDH detection was included as a loading control. (**B, D**) Relative expression of N, P, F, HN, and HMPV F protein in Vero (**B**) and A549 (**D**) cells from three independent infections. Protein expression was normalized to GAPDH and shown as fold-change compared with B/HPIV3 (lane 9). Expression of the HMPV F protein is shown relative to the HMPV wt F expressed by B/HPIV3 (lane 1). (**E**) Analysis of protein levels in the sucrose-purified viruses. Viruses grown in LLC-MK2 cells were purified by ultracentrifugation. The virions were lysed in RIPA buffer, total protein concentrations were quantified, and 1 µg per lane was used for Western blot analysis with specific antibodies (Materials and Methods). (**F**) Relative amounts of viral proteins in purified virions. B/HPIV3 P, F, and HN levels were normalized to N and shown as fold-change compared with those of the empty B/HPIV3 (lane 9) from the same experiment. Relative amounts of HMPV F protein (including F_0_ and F_1_ fragments) are depicted relative to that of the HMPV wt F expressed by B/HPIV3 (lane 1). (**B, D, F**) Statistical differences compared with B/HPIV3 (lane 9) or, for HMPV F graphs, compared with B/HPIV3 expressing wt HMPV F (lane 1), are indicated at the top of each graph. For HMPV F, pairwise differences between BBopt and GSopt versions are indicated as well.* *P* < 0.05, **P < 0.01, ***P < 0.001, ns – not significant (*P* > 0.05) by one-way ANOVA and Dunnett’s multiple-comparisons test. (**B–F**) The x-axis labels (1–8) correspond to the lane numbers and virus constructs 1–8 indicated on the top of A.

To evaluate the expression of HMPV F and B/HPIV3 proteins, Vero and A549 cells were infected at an MOI of 3 PFU per cell with the B/HPIV3/HMPV-F constructs, B/HPIV3 without insert, or mock-infected in medium without trypsin. Cells infected with HMPV A in the presence of added trypsin were included as a positive control for HMPV F expression. Cell lysates were prepared 48 h after infection and analyzed by Western blotting under reducing and denaturing conditions (Fig. 3A through D). Expression of all eight versions of HMPV F was detected in both cell lines. The inactive F_0_ precursor of the HMPV F protein is cleaved by trypsin-like proteases to yield the disulfide-linked F_1_ and F_2_ subunits (apparent molecular weight: about 60 and 47 kDa, respectively). Both the F_0_ precursor and the F_1_ subunit were detected in the HMPV A infected cells (grown in the presence of added trypsin) using a monoclonal antibody specific to HMPV F (Fig. 3A and C, lane 10). In cells infected with B/HPIV3 expressing HMPV F and grown without added trypsin, different cleavage patterns of HMPV F were observed for the native (wt, BBopt, GSopt) F and TMCT versions (Fig. 3A and C, lanes 1–4) as compared with the stabilized pre-F forms (Fig. 3A and C, lanes 5-8). As described in Materials and Methods, the HMPV F_0_ proteins encoded by constructs 1–4 contain the naturally occurring HMPV F cleavage site RQSR (aa 99-102), which requires exogenous trypsin for cleavage in Vero or A549 cells, while constructs 5–8 contain a modified cleavage site (RRRR), which is cleavable by the ubiquitous cellular furin-like proteases. Therefore, F_0_ forms of constructs 1–4 remained uncleaved in the absence of trypsin and were detected as F_0_ only, as expected, while F_0_ precursors of constructs 5–8 were cleaved into F_1_ and F_2_ subunits, of which F_0_ and F_1_ were detected by the monoclonal antibody (Fig. 3A and C, compare lanes 1–4 and 5–8). Quantitative comparison of protein expression in both cell types from three independent experiments showed that expression of the HMPV F protein by the GSopt constructs (lanes 3, 4, 6, and 8) was similar to that of wt F (Fig. 3B and D, panel HMPV F, lane 1). Expression of the BBopt HMPV F (lanes 2, 5, and 7) was slightly (lane 2) or significantly (lanes 5 and 7) reduced compared with wt F (lane 1). Both GSopt HMPV pre-F versions (lanes 6 and 8) expressed significantly more protein compared with their BBopt counterparts (lanes 5 and 7) (Fig. 3B and D). Compared to wt HMPV F (lane 1), GSopt codon optimization did not significantly increase HMPV F expression, but the BBopt optimization reduced the amount of HMPV F expressed during infection.

Given the sequential transcription of the parainfluenza virus genome, the insertion of an additional gene encoding HMPV F between the B/HPIV3 N and P genes is expected to decrease the transcription of BPIV3 P, M, and L and HPIV3 F and HN genes compared with B/HPIV3 without insert. To assess this, we evaluated the expression of BPIV3 N and P and the HPIV3 F and HN proteins using rabbit hyperimmune sera. Compared with B/HPIV3 control (lane 9), the amounts of BPIV3 N and P proteins in both Vero and A549 cells were reduced for most of the viruses except those expressing the native HMPV F (wt and BBopt, lanes 1 and 2), and except for B/HPIV3 expressing GSop-N46V/T160F (lane 8) in Vero cells. Expression of the HPIV3 F and HN proteins was also greatly reduced (Fig. 3B and D) for the B/HPIV3 vectors containing HMPV F insert. The GSopt-TMCT construct had a greater reduction in the global expression of the PIV3 proteins compared with the other constructs, which was consistent with, and might be the basis for, its greatly reduced plaque size and reduced replication compared to other viruses. However, despite this, it expressed HMPV F protein at amounts comparable to that of wt F and other GSopt versions.

### Protein packaging into the virions

Next, we evaluated if the HMPV F protein was incorporated into the B/HPIV3 particles. LLC-MK2 cells were infected with B/HPIV3 expressing various forms of HMPV F. As a control, cells were infected with HMPV A in the presence of trypsin. Virus particles released into the culture supernatant were purified by sucrose gradient ultra-centrifugation, and 1 µg total protein per virus was analyzed by Western blotting. None of the HMPV F versions, including GSopt-TMCT, were efficiently incorporated into the B/HPIV3 virions, as these contained 10–40-fold lower amounts of HMPV F protein (lanes 1–8) (Fig. 3E and F panel HMPV F) than HMPV particles (lane 10). The relative abundance of HMPV F protein detected in the B/HPIV3 particles closely reflected the expression patterns observed in the infected Vero and A549 cells, with GSopt versions detected at higher levels than the BBopt counterparts. This shows that HMPV F with or without BPIV3 F TMCT was not actively packaged by B/HPIV3; we speculate that the small amounts detected might represent membrane debris and/or extracellular vesicles, co-purified with the virions. Quantitative comparison showed that all versions of B/HPIV3/HMPV-F virions contained significantly less P protein than the B/HPIV3 control. Interestingly, even though HMPV F was not packaged efficiently, the GSopt versions had lower amounts of HPIV3 F and HN incorporated into their virions (Fig. 3F). This could be due to a higher HMPV F expression levels in the cells that may have competitively affected HPIV3 F and HN synthesis, processing, and/or transport to the plasma membrane, thereby reducing their packaging into the particle.

### Replication and immunogenicity of candidate vaccine viruses in hamsters

**Fig 4:**
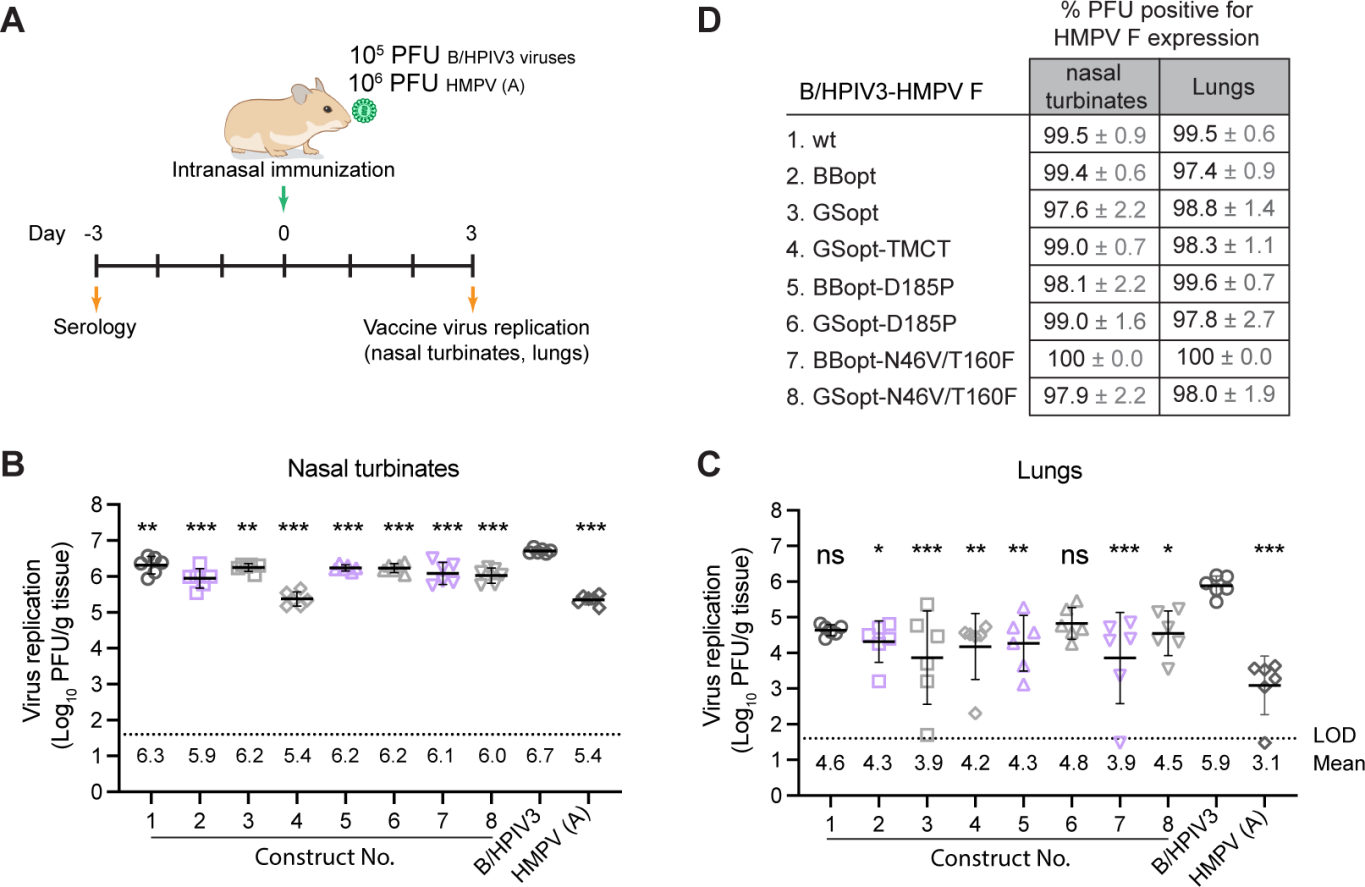
Replication of vaccine candidates in hamsters. (**A**) Timeline of the animal experiment and sample collection. Groups (*n* = 6) of 6-week-old golden Syrian hamsters were inoculated IN with 10^5^ PFU of B/HPIV3-HMPV F viruses. The B/HPIV3 empty vector (10^5^ PFU) and HMPV (CAN97-83) (10^6^ PFU) were included as controls. Three days after inoculation, the animals were euthanized, and lungs and nasal turbinates were collected. (**B, C**) Viral titers in the nasal turbinate (**B**) and lung (**C**) homogenates were determined by dual-staining immunoplaque assay. Individual animal titers (log_10_ PFU/g of tissue) are shown by symbols, and group means (±SD) are depicted as horizontal lines and numerically shown below the dotted line. The limit of detection (LOD) was 50 PFU/g of tissue. The x-axis labels (1–8) correspond to the viruses listed to the left of panel D. (**D**) The percentage (±SD) of dual-stained plaques in the nasal turbinate and lung homogenates indicating stability of HMPV F protein expression by the B/HPIV3 vectors. *P < 0.05, **P < 0.01, ***P < 0.001 by one-way ANOVA and Dunnett’s multiple-comparisons test.

Vaccine virus replication and stability of HMPV F expression were evaluated in golden Syrian hamsters. Groups of six animals were inoculated intranasally (IN) with B/HPIV3 vectors expressing HMPV F (constructs 1–8), B/HPIV3 control without insert, or recombinant HMPV CAN97-83 (subgroup A2). On day 3 after inoculation, nasal turbinates and lungs were collected (Fig. 4A), and clarified tissue homogenates were prepared. Virus titration was performed by dual-staining immunoplaque assay, simultaneously detecting PIV3 and HMPV F antigens, to determine virus titers and assess the stability of HMPV F expression during replication *in vivo*. As previously reported (30, 31), B/HPIV3 without insert replicated to high titers in the nasal turbinates and lungs (6.7 and 5.9 log_10_ PFU/g of tissue, respectively) (Fig. 4B and C). However, all B/HPIV3 vectors expressing HMPV F had 2- to 5-fold lower titers detectable in the nasal turbinates (Fig. 4B); the greatest reduction was observed for B/HPIV3 expressing GSopt-TMCT that had a 20-fold lower mean titer than the B/HPIV3 control. In the lungs, all candidate vaccine viruses reached 10–30-fold lower titers than the B/HPIV3 control (Fig. 4C), though these reductions were not statistically significant for B/HPIV3 expressing wt or GSopt-D185P HMPV F (constructs 1 and 6). All candidate vaccine viruses stably expressed HMPV F in the nasal turbinates and lungs, with 97.6%–100% and 97.4%–100% PFUs positive for HMPV F, respectively (Fig. 4D).

**Fig 5:**
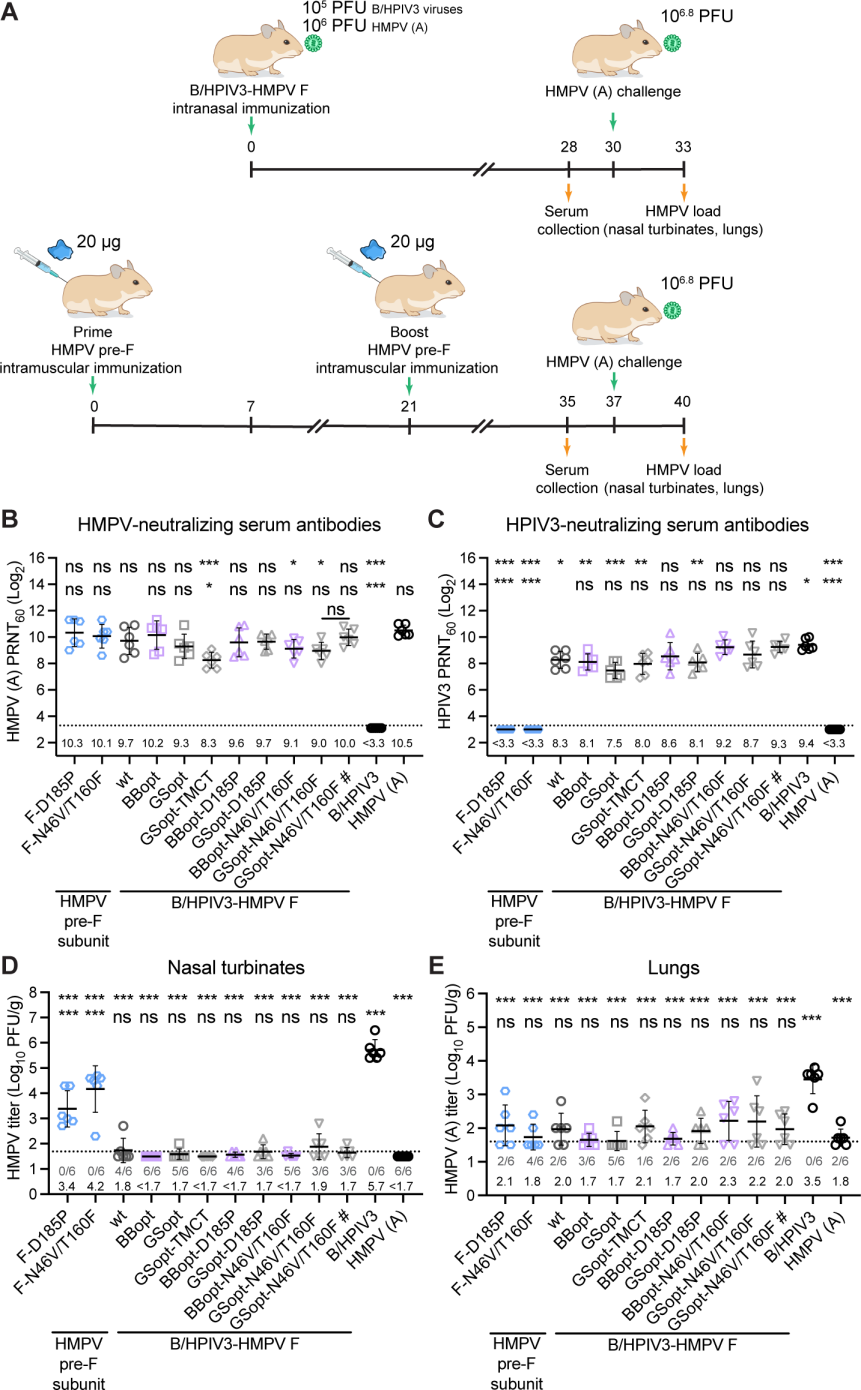
Immunogenicity and protective efficacy in hamsters. (**A**) Schematic of the animal study protocol. Groups of six golden Syrian hamsters received either a single IN dose of 10^5^ PFU of one of the eight B/HPIV3-HMPV F viruses or two IM doses of one of the soluble HMPV pre-F subunits F-D185P or F-N46V/T160F (20 µg per dose, given with alum-85 adjuvant) by IM injection 3 weeks apart. B/HPIV3 without insert (10^5^ PFU) and HMPV A (10^6^ PFU), administered IN, were included as controls. Sera were collected from all groups at 2 weeks post IM boost with HMPV pre-F subunit-vaccine and 4 weeks post IN immunization with indicated viruses to assess the serum virus-neutralizing antibody response. Three days later, hamsters were challenged IN with HMPV A (10^6.8^ PFU); lungs and nasal turbinates were collected 3 days post-challenge to examine HMPV replication by plaque assay. (**B, C**) Serum samples from immunized hamsters were analyzed for neutralizing antibodies against HMPV A (**B**) and HPIV3 (**C**); PRNT_60_ on Vero cells are shown. (**D, E**) HMPV challenge virus titers in the nasal turbinate (**D**) and lung (**E**) homogenates 3 days after challenge. Individual animal titers (log_10_ PFU/g of tissue) are shown by symbols, and group means (±SD) are depicted as horizontal lines and numerically above the x axis. The number of animals out of six with undetectable virus titers is shown below the dotted line. The limit of detection (LOD) was 50 PFU/g of tissue. *P < 0.05, **P < 0.01, ***P < 0.001, ns – not significant (*P* > 0.05) by one-way ANOVA and Dunnett’s multiple-comparisons test. PRNT_60_, 60% plaque reduction neutralization titer.

To evaluate the immunogenicity of candidate vaccine viruses, additional groups of six hamsters each were immunized IN with a single dose of each virus or with one of the two purified HMPV stabilized pre-F subunit proteins (F-D185P or F-N46V/T160F), mixed with alum-85 and administered IM as two doses 3 weeks apart (Fig. 5A). Serum samples were collected from all animals at 4 weeks after virus immunization or 2 weeks after the second IM dose of the HMPV pre-F subunit proteins. To assess the serum HMPV- and HPIV3-neutralizing antibody response, 60% plaque reduction neutralization (PRNT_60_) titers were determined on Vero cells. Animals immunized with 10^5^ PFU of B/HPIV3 expressing HMPV F developed serum HMPV A-neutralizing antibody titers comparable to those elicited by wild type HMPV A given at a 10^6^ PFU dose (Fig. 5B, statistics shown at the top of the panel [top line]), except for viruses expressing GSopt-TMCT (construct 4, *P* < 0.001), BBopt-N46V/T160F (construct 7, *P* = 0.03), and GSopt-N46V/T160F (construct 8, *P* = 0.01), which elicited significantly lower antibody titers. Animals immunized with B/HPIV3/GSopt-N46V/T160F#, a B/HPIV3/GSopt-N46V/T160F stock recovered and amplified in Vero cells, had slightly—but not significantly—higher antibody titers compared with the group immunized with the LLC-MK2 grown stock, and these titers more closely resembled those elicited by HMPV A. When compared with B/HPIV3 expressing wt HMPV F, B/HPIV3 vectors expressing the modified forms of HMPV F were similarly immunogenic, except for GSopt-TMCT (construct 4) that elicited a significantly lower antibody response (Fig. 5B, statistics shown at the top of the panel [bottom line]). This suggested that codon optimization, TMCT modification, or pre-F stabilizing mutations, did not improve HMPV F immunogenicity in hamsters. Two IM doses of the subunit HMPV pre-F proteins F-D185P or F-N46V/T160F induced serum antibody titers similar to those induced by their respective BBopt and GSopt versions expressed by B/HPIV3 given as a single IN dose.

Sera from animals immunized with the B/HPIV3 vectors expressing HMPV F were also analyzed for their neutralizing activity against HMPV subgroup B (strain CAN98-75). Since HMPV B replicates less efficiently and produces very small plaques on Vero cells as compared with HMPV A (Fig. S3A), the HMPV B 60% plaque reduction neutralization assay was optimized for LLC-MK2 cells using an overlay containing 2% TrypLE (Fig. S3B). The different versions of HMPV F expressed by B/HPIV3 induced high titers of serum HMPV B-neutralizing antibodies, albeit at reduced levels than those of animals immunized with HMPV A (Fig. S3C).

The hamster sera were also analyzed for the HPIV3-neutralizing antibodies. As expected, immunization with the HMPV subunit F proteins and HMPV A inoculation did not induce an antibody response to HPIV3 (Fig. 5C). BBopt-D185P, BBopt-N64V/T160F, GSopt-N64V/T160F, and GSopt-N46V/T160F# induced the strongest antibody response to HPIV3, which was comparable to that of the B/HPIV3 control, while the titers of the remaining candidates were lower (Fig. 5C, statistics shown at the top of the panel [top line]). There were no significant differences in the HPIV3-neutralizing antibody titers elicited by B/HPIV3 expressing modified versions of HMPV F compared to that expressing wt F (Fig. 5C, statistics shown at the top of the panel [bottom line]). Thus, all vaccine candidates induced a potent neutralizing antibody response to both HMPV and HPIV3.

### Protective efficacy against HMPV challenge in hamsters

To assess the level of protection against HMPV infection, hamsters were challenged IN with 10^6.8^ PFU of HMPV subgroup A (strain CAN97-83) 30 days after intranasal immunization with B/HPIV3 vectors expressing HMPV F or 16 days after the second IM dose of the HMPV pre-F subunit protein vaccines (Fig. 5A). On day 3 post-challenge, nasal turbinates and lungs were collected, and replication of challenge HMPV was quantified by titration of the clarified tissue homogenates using an HMPV immunoplaque assay (Fig. 5D and E). Following the challenge, HMPV replicated to high titers (mean: 10^5.7^ PFU/g of tissue) in the nasal turbinates of animals immunized with B/HPIV3 control (Fig. 5D). Compared with these animals, all other groups showed a significant reduction in challenge virus replication (Fig. 5D, statistics shown at the top of the panel [top line]), indicating that all B/HPIV3 candidates expressing HMPV F, regardless of its modifications, were highly protective at levels similar to those of an HMPV A infection (Fig. 5D, statistics shown at the top [bottom line]). No infectious challenge virus was detected in the nasal turbinates of most or all animals in each group, except for a few with very low titers, indicating a reduction in challenge virus replication by around 10,000-fold compared with the B/HPIV3-immunized animals. In contrast, animals immunized by the IM route with two doses of the recombinant adjuvanted HMPV pre-F proteins showed only moderate protection in the nasal turbinates. Their challenge HMPV titers were significantly higher than those of animals immunized with HMPV or with B/HPIV3 candidates expressing HMPV F. Thus, B/HPIV3 vectors expressing only the HMPV F protein provided protection in the upper respiratory tract against HMPV challenge comparable to that elicited by an HMPV infection, which induces an immune response against the full array of HMPV antigens. Intramuscular immunization with HMPV pre-F antigens was much less protective against challenge virus replication in the nasal turbinates.

The results were less clear in the lungs due to an overall lower level of challenge virus replication, even in the HMPV-naïve control group immunized with B/HPIV3 without an insert. Nevertheless, all animals immunized with the B/HPIV3 vectors expressing HMPV F had significantly lower HMPV challenge virus titers in the lungs compared with the B/HPIV3 control group (Fig. 5E, statistics shown at the top [top line]). Importantly, the protection provided by the vaccine candidates in the lungs was similar to that provided by wt HMPV A immunization (Fig. 5E, statistics shown at the top [bottom line]). In contrast to the nasal turbinates, the recombinant adjuvanted HMPV pre-F subunits (F-D185P and F-N46V/T160F) provided protection in the lungs, reducing the HMPV titers to levels comparable to those of the candidate vaccine viruses and wt HMPV A. This suggested that the high serum HMPV-neutralizing antibody titers induced by the pre-F subunit vaccines were more protective in the lungs than in the nasal turbinates.

We selected B/HPIV3/GSopt-N46V/T160F for further evaluation in Phase 1 studies based on the superior properties of the N46V/T160F stabilized pre-F subunit antigen (23), such as efficient expression, improved thermostability, and immunogenicity as a protein subunit vaccine. These attributes were further supported by its immunogenicity and stable expression from B/HPIV3 in the present study. Thus, the Vero cell generated P2 stock of this candidate, i.e., B/HPIV3/GSopt-N46V/T160F#, was further characterized as described below.

### HMPV F (GSopt-N46V/T160F) does not mediate infectivity of B/HPIV3/GSopt-N46V/T160F#

As described above, none of the HMPV F versions were efficiently incorporated into the B/HPIV3 particle and are therefore unlikely to mediate B/HPIV3 infection. To experimentally confirm this, we tested the infectivity of B/HPIV3/GSopt-N46V/T160F# in Vero cells in the presence of HMPV- and/or HPIV3-neutralizing antibodies. Viruses, including B/HPIV3/GSopt-N46V/T160F#, recombinant HPIV3 expressing green fluorescent protein (rHPIV3-GFP), recombinant (r)HMPV CAN97-83 (HMPV subgroup A), and HMPV CAN98-75 (HMPV subgroup B), were included in virus neutralization assays using antisera raised in specific pathogen-free rabbits against HPIV3, HMPV subgroups A, or subgroup B. The HPIV3 antiserum is a well-characterized control known to neutralize HPIV3 but not HMPV. The HMPV A and B specific sera neutralize HMPV A and B strains at different levels of potency, reflecting antigenic differences between the subgroups, but do not neutralize HPIV3.

As shown in Table 1, B/HPIV3/GSopt-N46V/T160F# is effectively neutralized by the HPIV3 antiserum but not by the HMPV A or B specific sera, indicating this virus was sensitive to HPIV3- but not to the HMPV A- or B- neutralizing antibodies. The neutralizing titers of the HPIV3 antiserum obtained with B/HPIV3/GSopt-N46V/T160F# differed by only less than two-fold from those against the rHPIV3-GFP-positive control virus. These results are within the range of variability of a biological assay performed on two different virus stocks. The addition of HMPV A or B specific sera to HPIV3-neutralizing serum did not substantially alter the potency of the HPIV3 antiserum to neutralize B/HPIV3/GSopt-N46V/T160F# or the rHPIV3-GFP positive control, indicating no additive effect of HMPV neutralizing antibodies on neutralization of B/HPIV3/GSopt-N46V/T160F#. As expected, the HMPV A and B antisera neutralized HMPV A and B, but not B/HPIV3/GSopt-N46V/T160F# or rHPIV3-GFP, with about eight-fold higher neutralizing titers obtained for each virus with the respective serum to the homologous subgroup. The HPIV3 antiserum did not neutralize HMPV. These results confirm that infectivity of B/HPIV3/GSopt-N46V/T160F# vaccine candidate relies on the HPIV3 HN and F proteins and that the HMPV F protein expressed by this virus does not contribute to infectivity.

**TABLE 1:** B/HPIV3/GSopt-N46V/T160F# is neutralized by HPIV3- but not the HMPV-neutralizing antibodies^*a*^

Serum	PRNT_60_ (reciprocal log_2_) to the indicated virus			
	B/HPIV3/GSopt-N46V/T160F#	rHPIV3-GFP	rHMPV CAN97-83 (A)	HMPV CAN98-75 (B)
HPIV3 serum	10.2	11	<3.3	<3.3
HMPV A serum	<3.3	<3.3	11	5.9
HMPV B serum	<3.3	<3.3	7.9	8.9
HPIV3 + HMPV A serum	10.2	11.1	10.5	6.1
HPIV3 + HMPV B serum	10.2	11.2	8.3	8.7

^*a*^ 60% plaque reduction neutralization assays were performed on Vero cells in which about 20–70 plaque-forming units of B/HPIV3/GSopt-N46V/T160F#, rHPIV3-GFP, HMPV CAN97-83 (A), or HMPV CAN98-75 (B) were incubated with fourfold serial dilutions of the indicated rabbit hyperimmune sera raised in specific pathogen-free rabbits against sucrose-purified HPIV3, HMPV CAN97-83 (subgroup A) (32), or HMPV CAN98-75 (subgroup B). After neutralization for 1 h at 37°C, virus/serum mixtures were added to sub-confluent monolayers of Vero cells, grown in 24-well plates. After a 1 h adsorption at 37°C, 0.8% methyl cellulose was added. Plates containing PIV3 or HMPV were incubated at 32°C or 37°C, 5% CO_2_, and were fixed after 6 or 7 days, respectively. Plaques were visualized by immunostaining, and the PRNT_60_ was determined (Materials and Methods) for each virus/serum combination. The limit of detection of the PRNT_60_ assay was 3.3 log_2_. rHMPV, recombinant HMPV; PRNT_60_, 60% plaque reduction neutralizing titer.

### Temperature sensitivity of B/HPIV3/GSopt-N46V/T160F

**Fig 6:**
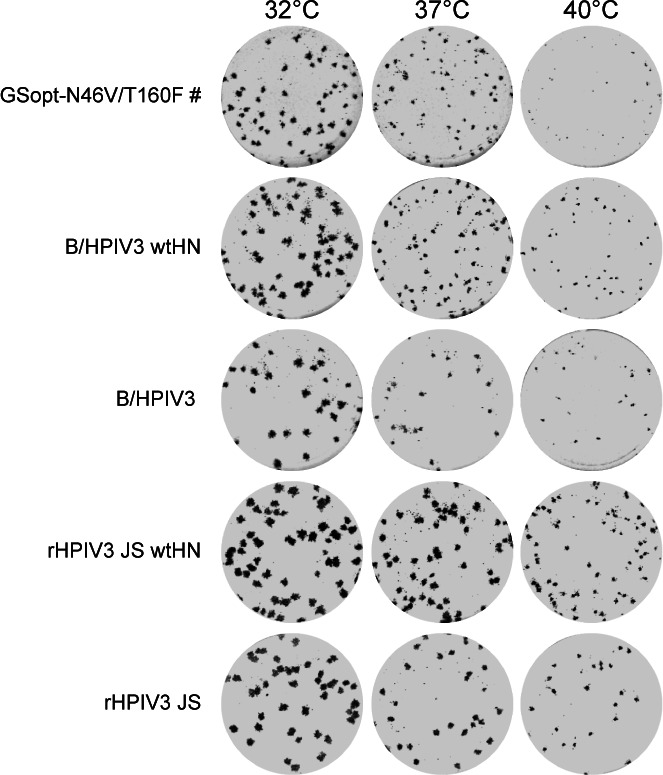
B/HPIV3/GSopt-N46V/T160F# plaque formation is temperature-sensitive and is restricted by increased temperature in A549 cells. The plaque phenotype of B/HPIV3/GSopt-N46V/T160F# was evaluated in human lung epithelial A549 cells, incubated at 32°C, 37°C, or 40°C. Cells seeded in 24-well plates were inoculated with serial dilutions of indicated virus stocks. Following adsorption, an L-15 or F-12K medium overlay containing 0.8% methylcellulose was added, and plates were incubated at the indicated temperatures in temperature-controlled water baths or tissue culture incubators, respectively. On day 7, cells were fixed, and plaques were visualized by immunostaining. A representative PIV3 plaque immunostaining of cells from tissue culture incubators is shown (grayscale) for the virus dilutions appropriate to visualize plaque sizes.

Live-attenuated respiratory virus vaccine candidates with temperature-sensitivity (ts) phenotypes are expected to be restricted in replication at the higher physiological temperatures of the lower respiratory tract, limiting the potential for reactogenicity. To test for a possible ts phenotype, we evaluated the replication of B/HPIV3/GSopt-N46V/T160F# and control viruses at different temperatures by plaque assay on A549 and LLC-MK2 cells. A version of B/HPIV3 deemed well-tolerated and immunogenic as a live intranasal HPIV3 vaccine candidate in a small Phase 1 study in HPIV3 seronegative infants and children, 6–36 months of age, was included as a control. This B/HPIV3 control represents a benchmark and serves as a reference and bridging control for subsequent studies of B/HPIV3-based vaccine candidates in the HPIV3-seronegative target population (25, 33). It differs from the B/HPIV3 vector in the present study only by two amino acid assignments in the HN ORF, bearing non-wt marker assignments 263I and 370T (Materials and Methods). Recombinant HPIV3 strain JS (rHPIV3 JS) bearing marker assignments 263I and 370T in HN and HPIV3 with the wt HN amino acid assignments 263T and 370P (rHPIV3 JS wtHN), matching those of the B/HPIV3 vector (B/HPIV3-wtHN) used in the present study, were included as additional controls. A549 or LLC-MK2 cells, seeded in 24-well plates, were incubated with serial dilutions of indicated virus stocks in closed stainless-steel containers, submerged in temperature-controlled water baths or kept in tissue culture incubators. On day 7 (A549) or 8 (LLC-MK2), cells were fixed with 80% methanol, and plaques were visualized by immunostaining as described above. rHPIV3 JS and rHPIV3 JS wtHN readily formed plaques in both cell lines at temperatures ranging from 32°C to 40°C, with no reduction in titers at higher temperatures (Table 2; Table S1). In A549 cells, a small-plaque phenotype was observed for B/HPIV3 and its derivatives at 37°C, the physiological temperature of the human lower respiratory tract, and B/HPIV3/GSopt-N46V/T160F# formed microplaques at 40°C (Table 2; Fig. 6). In LLC-MK2 cells, B/HPIV3 and B/HPIV3-wtHN formed small plaques at 39°C, and B/HPIV3/GSopt-N46V/T160F# formed small plaques at 38°C; and all three formed microplaques at 40°C (Table S1). The identity of the 263 and 370 amino acid assignments had no effect on the ts phenotype.

**TABLE 2:** Temperature sensitivity and plaque phenotype of B/HPIV3/GSopt-N46V/T160F# in A549 cells

Virus^*b*^	Virus titer (log_10_ PFU/mL) at temperature (°C)^*a*^			Lowest restrictive temperature		
	32	37	40	T_SP_^*c*^	T_MP_^*d*^	T_SH_^*e*^
rHPIV3 JS	8.3	8.2	8.2	40	>40	>40
rHPIV3 JS wtHN	8.5	8.5^*c*^	8.4^*c*^	40	>40	>40
B/HPIV3	6.6	6.5^*c*^	6.3^*c*^	37	>40	>40
B/HPIV3-wtHN	7.3	7.3^*c*^	6.9^*c*^	37	>40	>40
B/HPIV3/GSopt/N46V/T160F#	6.8	6.7^*c*^	6.3^*d*^	37	40	>40

^*a*^ The temperature sensitivity phenotype for each virus was evaluated by plaque assay in A549 cells at the indicated temperatures.

^*b*^ B/HPIV3 represents a reference control of a vaccine candidate that was attenuated in HPIV3 seronegative infants and children (25, 33). rHPIV3 JS and rHPIV3 JS wtHN, as well as B/HPIV3 and B/HPIV3-wtHN, differ only at the HN amino acid assignments 263 and 370 (Materials and Methods).

^*c*^ T_SP_ (small-plaque temperature) is defined as the lowest restrictive temperature at which the small-plaque phenotype is observed.

^*d*^ T_MP_ (micro-plaque temperature) is defined as the lowest restrictive temperature at which the micro-plaque phenotype is observed.

^*e*^ T_SH_ (shutoff temperature) is defined as the lowest restrictive temperature at which the reduction in titer compared with that at 32°C is 100-fold or greater than that observed for the wild-type virus at the two temperatures.

Thus, compared with HPIV3 JS, B/HPIV3 and derivatives were slightly restricted in replication at higher temperatures. The presence of an additional HMPV gene further reduced the plaque size compared with B/HPIV3-wtHN or the previous B/HPIV3 benchmark vaccine candidate that was well-tolerated and immunogenic in HPIV3-seronegative children (25). Based on these data, B/HPIV3/GSopt-N46V/T160F# is predicted to be at least as attenuated in humans as B/HPIV3.

In summary, B/HPIV3/GSopt-N46V/T160F# and B/HPIV3 vectors expressing other forms of HMPV F were immunogenic against HPIV3 and HMPV A and B. In hamsters, these candidates were as protective against HMPV A challenge as a wt HMPV infection, even though they expressed only a single HMPV antigen and were given at a 10-fold lower dose than wt HMPV A. A single intranasal dose provided greater protection in the upper respiratory tract against HMPV replication and shedding than the two IM doses of the adjuvanted HMPV pre-F subunit antigens F-D185P or F-N46V/T160F.

## DISCUSSION

Pediatric HMPV and HPIV3 infections cause severe and sometimes life-threatening disease in children under 5 years of age (1–3, 9) and a single-dose vaccine that could protect against both would be desirable. To achieve bivalency, we expressed the HMPV F protein from B/HPIV3. B/HPIV3 has been evaluated as a pediatric intranasal HPIV3 vaccine in previous studies and was found to be sufficiently attenuated, well-tolerated, and immunogenic in infants and young children (25, 26). We have also previously used this vector to express RSV F and SARS-CoV-2 S proteins for dual protection against HPIV3/RSV (27) and HPIV3/SARS-CoV-2 (34, 35). B/HPIV3 expressing the F protein of HMPV sublineage A1 has been studied in hamsters and elicited protective immunity in African green monkeys (36, 37). However, the *in vivo* stability of the HMPV F insert expression was not evaluated. Moreover, recent prevalence studies have reported that HMPV sublineage A1 is detected less frequently than other genotypes in most countries. It was not observed in the Netherlands after 2006 (38), and many recent studies have failed to detect HMPV A1, indicating that this genotype might have become extinct (39). Here, we used the HMPV F coding sequence from strain CAN97-83 (subgroup A2) and constructed eight B/HPIV3 derivatives expressing HMPV wt F or its modified versions to identify the most immunogenic candidate vaccine.

Our earlier studies with B/HPIV3 expressing RSV F revealed that the immunogenicity and protective efficacy of the RSV F protein are increased modestly by higher protein expression due to codon optimization and greatly by stabilization of RSV F in the pre-F conformation (27, 29, 30). Therefore, we evaluated two codon-optimized HMPV F ORFs and compared their expression with that of the wt ORF. Codon optimization did not yield a significant increase in HMPV F expression compared with that of wt ORF by Western blotting, but a stronger HMPV F immunostaining by plaque assay suggested higher expression for the GSopt codon optimized ORF. However, this was not associated with a detectable increase in immunogenicity in hamsters. Reduced expression of the B/HPIV3 N, P, F, and HN proteins was observed for most of the B/HPIV3 vectors containing an HMPV F insert. Insertion of a foreign gene into a paramyxovirus genome can reduce replication, transcription, and viral protein synthesis, a likely outcome of increased genome size. Insertion of a supernumerary gene also causes a shift in the position of vector genes away from the genome promoter, which reduces their expression. This is due to the known 3′−5′ polar gradient of transcription for negative-strand RNA viruses—the genes further downstream from the 3′ genomic promoter are transcribed at reduced levels. HMPV F insertion had minimal to no effect on the final titers of virus stocks, but we observed a slight delay, although statistically insignificant, in replication to peak titers (growth curves). Thus, the reduced expression of the B/HPIV3 proteins could be due to the slightly slower replication and/or a shift in gene positions.

The TMCT mutation in HMPV F was also introduced based on our prior experience with RSV F, for which a TMCT modification significantly increased its packaging in the B/HPIV3 particle (27), induced antibodies against site Ø (a potent RSV neutralization epitope (40), conferred greater titers of high-quality antibodies that neutralized RSV in the absence of complement, and resulted in a dramatic increase in immunogenicity and protective efficacy (27). However, none of the effects seen for RSV F were observed for the GSopt-TMCT version of HMPV F. This virus had reduced HMPV immunogenicity, which could be attributed to a possibly altered HMPV F folding and loss of immunogenic epitopes due to TMCT mutation. We speculate that altered folding also might have obscured the recognition of BPIV3 F TMCT domains by the vector proteins responsible for packaging, leading to a lack of incorporation into the particle. Although GSopt-TMCT HMPV F was expressed in cell culture at levels similar to wt HMPV F or other GSopt versions, reduced replication of the vector, almost by 10-fold, in the nasal epithelium of hamsters may have decreased the total HMPV F antigen load, also contributing to reduced immunogenicity.

Adsorption of human sera with the RSV pre-F, but not the post-F, protein removes most of the RSV-neutralizing activity, indicating that the RSV-neutralizing antibodies are predominantly directed towards the unique and potently neutralizing epitopes, such as site Ø in the pre-F form (17, 19, 40). Consistently, the immunogenicity of an RSV F-based vaccine is immensely improved by stabilization of F in the pre-F conformation (27, 40), and therefore, all recently licensed adult RSV vaccines are based on the pre-F form (41). Being closely related, similar studies were performed for the HMPV F protein. However, in initial studies, each of the pre- and post-F forms of HMPV effectively adsorbed and removed most of the HMPV-neutralizing activity from human sera (19), indicating that both conformations induce HMPV-neutralizing antibodies and contribute to protection. In recent studies, next-generation pre-F-stabilized forms of HMPV F protein were developed (20–23), showing improved thermostability. They represent promising subunit vaccine candidates, with antigenic epitopes that include the pre-F-exclusive sites Ø, III, and V. In animal models, these exceeded the immunogenicity and efficacy of post-F when evaluated as injectable subunit vaccines usually requiring sequential administration of two adjuvanted doses. However, non-replicating inactivated HPIV3 vaccines are associated with markers of priming for enhanced disease in experimental animals (42) and are contraindicated for infants and young children.

An intranasal vaccine would be desirable for effective protection of infants and young children against respiratory illness. In the case of influenza vaccines, live-attenuated IN vaccines had significantly better efficacy than inactivated vaccines among young children (43, 44). They are needle-free, easy to administer, noninvasive, and painless, making them ideal for pediatric use. A live-attenuated B/HPIV3 vaccine expressing a pre-F-stabilized HMPV F antigen would represent a single-dose vaccine against two important pediatric pathogens. Since the HMPV F protein is nonessential for the infectivity of B/HPIV3, it can be stabilized in a non-fusogenic pre-F conformation. Delivered IN, this vaccine will express HMPV pre-F in the respiratory mucosal cells, mimicking a natural HMPV infection for the stimulation of a protective immune response. We modified HMPV F by introducing N46V/T160F substitutions to increase its stability in the pre-F state, which was previously identified as the most immunogenic subunit antigen compared with post-F (23). We also included, as a control, a first-generation HMPV pre-F antigen stabilized by D185P substitution (19). These pre-F proteins encoded by the GSopt ORFs were expressed by B/HPIV3 in greater abundance compared with the BBopt ORFs *in vitro*, but this did not translate to greater immunogenicity or protection in hamsters. We found both N46V/T160F and D185P pre-F forms to be comparably immunogenic and protective when expressed by B/HPIV3 with immunogenicity similar to each other and to wt F.

A few possible explanations for this disparity between the vectored F proteins of RSV and HMPV include: (i) unlike RSV F, both pre- and post-F forms of HMPV F have potent neutralization epitopes as already noted, (ii) in the case of HMPV, the structure-based pre-F-stabilizing mutations may be less effective at stabilizing the pre-F structure than those of RSV F, (iii) the pre-F state of HMPV wt F might be more stable than that of RSV F, resulting in immunogenicity similar to that of stabilized pre-F, and/or (iv) the shared pre- and post-F antigenic sites I, II, and IV of HMPV F are strongly protective, potentially obscuring the added immunogenic benefits of stabilizing sites Ø, III, and V in HMPV pre-F antigens. It is also possible that the glycosylation at Asn172 at the apex of the HMPV pre-F protein masks antigenic site Ø, inhibiting the elicitation of site Ø specific antibodies (19, 45). While none of the HMPV F antigens was clearly superior in the B/HPIV3 background, B/HPIV3 expressing N46V/T160F was selected as the lead candidate based on the superior expression, thermostability, and immunogenicity of the corresponding subunit antigen (23).

It is important to highlight that, despite being given at a 10-fold lower dose than HMPV A and containing only a single HMPV antigen, most of the candidate vaccine viruses induced HMPV A-neutralizing antibody titers similar to those elicited by wt HMPV A. The hamster sera also contained high titers of HMPV B-neutralizing antibodies, as expected, due to the conservation and antigenic relatedness of the HMPV F proteins of the two subgroups (10, 12, 14). The titers were significantly higher for the wt HMPV A immunized animals than most of the B/HPIV3 vectored candidates.

The two subunit pre-F proteins, delivered IM, behaved very similarly in hamsters. Both stimulated high titers of serum HMPV-neutralizing antibodies and provided significant protection in the lungs and nasal turbinates, albeit their protective efficacy was much weaker in the nasal turbinates, allowing challenge virus replication at several orders of magnitude higher than in animals immunized IN with B/HPIV3 expressing the same proteins. This weak protection in the nasal turbinates is likely due to low concentrations of HMPV-neutralizing antibodies in the nasal lining fluid. Most adjuvants, including alum, are inefficient at establishing olfactory-tissue-resident antibody-secreting plasma cells and provide weak protection in the nasal tissues, despite inducing high titers of serum antibodies—which do not efficiently protect the nasal mucosa likely due to their exclusion by a proposed blood-endothelial barrier (46). However, mucosal immunization induces tissue-resident plasma cells that secrete mucosal antibodies and protect the nasal mucosa. This difference in efficacy between the subunit IM and B/HPIV3 IN vaccinations is remarkable and demonstrates that the HMPV F protein provides a superior protection when delivered mucosally by a replicating virus compared with parenteral administration as an adjuvanted subunit vaccine. Thus, the route of immunization (IN versus IM) and the nature of vaccine (replicating versus non-replicating) have a greater impact on an HMPV vaccine efficacy than the stabilization of HMPV F structure in various biologic forms.

We have previously shown that B/HPIV3 expressing the RSV F protein, when used as a booster following priming with an attenuated RSV vaccine given 2, 6, or 15 months earlier, replicated efficiently in the presence of RSV-specific antibodies and induced a strong secondary immune response in African green monkeys (47). In comparison, replication of an attenuated RSV booster in animals with pre-existing RSV immunity was highly restricted, resulting in a much weaker boost. Thus, a prime-boost strategy using a live-attenuated RSV vaccine in combination with a heterologous B/HPIV3 vectored boost provided a robust secondary response that was far superior to that achieved by multiple doses of an attenuated RSV. The B/HPIV3/GSopt-N46V/T160F# candidate vaccine virus, selected for phase I clinical evaluation, could be used as a primary HPIV3/HMPV vaccine in 6- to 24-month-old children. Alternatively, as a component of a prime-boost regimen, B/HPIV3/GSopt-N46V/T160F# could be administered as a booster following a primary immunization with a live-attenuated HMPV vaccine to achieve a robust anamnestic response.

In summary, the B/HPIV3/GSopt-N46V/T160F# candidate is highly immunogenic against HPIV3 and HMPV, is protective against HMPV in hamsters, and is predicted to be immunogenic against HPIV3 and HMPV in HPIV3-seronegative children. HMPV F with N46V/T160F substitutions is not functional for B/HPIV3 and does not contribute to its infectivity. In multiple clinical studies, B/HPIV3 itself and B/HPIV3 expressing RSV F given as live IN vaccines were well-tolerated and safe in infants and young children (25, 26). B/HPIV3/GSopt-N46V/T160F# is expected to be similarly safe in HPIV3-seronegative children and is currently being evaluated in a pediatric phase I study (ClinicalTrials.gov identifier NCT06546423).

## MATERIALS AND METHODS

### Cells and viruses

Vero cells (African green monkey kidney epithelial, ATCC CCL-81) and LLC-MK2 cells (rhesus macaque kidney epithelial, ATCC CCL-7) were cultured in Opti-MEM medium (ThermoFisher Scientific, Waltham, MA) containing 5% fetal bovine serum (FBS; Hyclone, Logan, UT). The BHK (baby hamster kidney) BSR-T7/5 cell line stably expressing T7 RNA polymerase was maintained as previously described (48). The culture medium was supplemented with 2% geneticin (ThermoFisher Scientific) at every other passage to maintain T7 transgene expression. Human lung epithelial A549 cells (ATCC CCL-185) were grown in F-12K medium (ATCC) containing 10% FBS, 1× L-Glutamine (ThermoFisher Scientific). B/HPIV3 was grown in LLC-MK2 cells. HMPV A (strain CAN97-83, GenBank accession no. AY297749) was propagated in Vero cells using Opti-MEM medium containing 2% TrypLE Select (ThermoFisher Scientific), without FBS. TrypLE Select was replenished on day 4. To generate virus stocks, monolayers were infected at a multiplicity of infection (MOI) of 0.01 PFU/cell, followed by incubation at 32°C. Culture supernatants were harvested on day 7, clarified by centrifugation, and stored at −80°C. Virus stocks were titrated in Vero cells by plaque immunostaining.

### Recombinant HMPV F expression and purification

Two pre-F forms of the HMPV F protein of strain CAN97-83 (subgroup A2) (GenBank accession AY145296), F-D185P and F-N46V/T160F, were recombinantly expressed in HEK293 cells (Sino Biological, Beijing, China) and purified by affinity and size exclusion chromatography (23). Each HMPV F protein was expressed from a plasmid and contained only the ectodomain (amino acids 1–490) with the pre-F stabilizing amino acid substitutions, D185P or N46V/T160F, and substitutions of the trypsin-dependent cleavage site (RQSR; amino acids 99–102) by a furin protease cleavage site (RRRR), followed by a short glycine linker, a T4 foldon trimerization domain, the human rhinovirus 3C protease cleavage site, a hexa-histidine tag, and a strep II tag at the C-terminus (23). Analysis of the purified proteins by biolayer interferometry to study binding with HMPV F-specific antibodies known to bind specifically to only pre-F, only post-F, or both pre- and post-F epitopes confirmed that both proteins were stable in pre-F conformation (23). These antigens were included in the hamster study as subunit protein vaccines, adjuvanted with alum-85, and administered by the intramuscular (IM) route.

### Design of B/HPIV3 expressing HMPV F protein

The cDNA clone of the B/HPIV3 antigenome was constructed previously (49). B/HPIV3 is a chimeric virus that consists of bovine PIV3 (BPIV3, Kansas strain, GenBank accession no. AF178654) in which the fusion (F) and hemagglutinin neuraminidase (HN) genes have been replaced with those from human PIV3 (JS strain; GenBank accession: Z11575). B/HPIV3 was modified by two amino acid substitutions in the HN protein (I263T and T370P) that removed two previously described sequence markers and restored the wild-type (wt) sequence (50). Eight B/HPIV3 constructs were designed to express the F protein of HMPV subgroup A2 (strain CAN97-83, GenBank accession no. AY297749) (Fig. 1A). Eight different ORFs were designed, including the wt ORF (construct 1) and seven variants (constructs 2-8). The F ORF was codon-optimized by Biobasic (Markham, ON) and Genscript (Piscataway, NJ), named as BBopt and GSopt (constructs 2 and 3), respectively, to increase F protein expression. In another construct called GSopt-TMCT (construct 4), the GSopt F ORF was modified by replacing its transmembrane and cytoplasmic tail (TMCT) domains (nt 1,474–1,620) with those of the BPIV3 F, to potentially enhance packaging into the vector particle, as previously described for the RSV F protein (27). In the next set of constructs (constructs 5–8), based on GSopt or BBopt F, we replaced the naturally occurring trypsin-dependent cleavage site RQSR (residues 99–102) with RRRR, a cleavage site found in the F protein of a closely related avian metapneumovirus type A. This site is recognized by furin-like proteases, eliminating the need for added trypsin to mediate cleavage during *in vitro* propagation. To increase the stability of HMPV F protein in the pre-F conformation, the GSopt and BBopt F ORFs were modified by amino acid substitutions D185P (constructs 5 and 6) as previously described (19), or N46V/T160F (construct 7 and 8) (23).

The synthetic DNAs were designed for insertion into the B/HPIV3 full-length cDNA using an *Asc*I site in the downstream noncoding region of the N gene preceding the N gene end signal (Fig. 1A, enlargement). To transcribe the HMPV F insert as a separate mRNA, all versions of HMPV F ORFs were designed to be preceded by the BPIV3 transcription signals including an N gene-end signal (AAGTAAGAAAAA), an intergenic region (CTT), and a gene-start signal (AGGATTAATGGA) (in the 5′ to 3′ order in the [+]-sense antigenome). *Asc*I sites were placed to flank each HMPV F insert, for insertion into the unique *Asc*I site in the antigenome. The sequences of the plasmid-borne antigenome cDNAs were fully confirmed by Sanger sequencing.

### Rescue and preparation of working stocks of recombinant viruses

All viruses were recovered by co-transfecting BHK BSR-T7/5 cells (48) with a T7 RNA polymerase driven set of expression plasmids, including the respective full-length virus antigenome plasmid together with the support plasmids expressing the BPIV3 N, P, and L proteins. At 48 h post-transfection, the transfected cells were co-cultured with a 50% confluent monolayer of LLC-MK2 cells and incubated at 32°C for 7 days, after which the culture supernatant containing the virus was clarified and stored at −80°C. This P1 stock was passaged once more in LLC-MK2 cells to obtain a P2 stock. Infected cell supernatants were clarified by centrifugation, snap-frozen on dry ice, and stored at −80°C. B/HPIV3/GSopt-N46V/T160F (construct 8, Fig. 1A) was also recovered in Vero cells by co-transfecting with the T7-driven expression plasmids indicated above, plus an additional plasmid designed to express T7 RNA polymerase under control of a CMV promoter. The resulting P1 stock was passaged once more in Vero cells to generate the P2 stock, termed B/HPIV3/GSopt-N46V/T160F#. Genome sequences of the recombinant viruses were confirmed by sequencing of the un-cloned overlapping RT-PCR products derived from viral RNA extracted from the P2 virus stocks.

### Stability of HMPV F expression by B/HPIV3

Virus stocks were evaluated for the stability of HMPV F expression by dual-antigen immunostaining plaque assay for simultaneous detection of HMPV F and B/HPIV3 antigens. Vero and A549 cells were infected with serially diluted viruses, overlaid with medium containing 0.8% methylcellulose, and incubated at 32°C for 7 days. The overlay for HMPV A plaque assay contained 4% or 0.5% of TrypLE in Vero and A549 cells, respectively. For staining, the cells were fixed with cold 80% methanol, blocked by incubation for 1 h with Odyssey blocking buffer (LICORbio, Lincoln, NE) at room temperature, and then incubated for 1 h at room temperature with a mixture of two HMPV F-specific human monoclonal antibodies (MPE8 and MPF5 [51, 52]) each at 1:2,500 dilution and a rabbit hyperimmune serum raised against sucrose-gradient-purified HPIV3 (MS456) at 1:5,000 dilution in Odyssey blocking buffer (LICORbio). Next, infrared dye-conjugated secondary antibodies of human and rabbit specificity (LICORbio) were used for detection. Plaques were visualized using the Odyssey infrared scanner (LICORbio) and were pseudo-colored to appear red and green for HMPV F and B/HPIV3 antigens, respectively. Plaques appearing yellow on superimposing red and green images indicated the expression of HMPV F protein by the B/HPIV3 vector.

### Multicycle replication in cell culture

A549 and Vero cells in six-well plates were infected in triplicate with the indicated viruses at an MOI of 0.1 or 0.01 PFU per cell. After adsorption for 3 h at 37°C, cells were washed to remove the virus inoculum, and 3 mL of fresh medium with 2% FBS was added. Cells were incubated at 32°C for 7 days. Every 24 h, 0.5-mL aliquots of culture medium were collected and snap-frozen on dry ice, and 0.5 mL of fresh medium was added to each well. Virus aliquots from each time point were titrated side-by-side on 24-well plates of Vero cells as described above.

### Analysis of viral protein expression by Western blotting

A549 or Vero cells in six-well plates were infected at an MOI of 3 PFU/cell with the indicated viruses and incubated at 32°C for 48 h. Cells were washed once with PBS and lysed with 300 µL LDS lysis buffer (ThermoFisher Scientific) containing NuPAGE reducing agent (ThermoFisher Scientific). Cell lysates were passed through a QIAshredder (Qiagen, Germantown, MD), heated for 10 min at 70°C, separated on 4 to 12% Bis-Tris NuPAGE gels (ThermoFisher Scientific) in the presence of antioxidant (ThermoFisher Scientific), and the resolved proteins were transferred to PVDF membranes. Following incubation with the blocking buffer (LICORbio) for 1 h at room temperature, membranes were incubated overnight with the primary antibodies at 4°C. HMPV F protein was detected by a hamster monoclonal antibody mAb 1017 (53), BPIV3 N and P proteins by a rabbit polyclonal hyperimmune serum against purified HPIV3 (29), HPIV3 HN and F were individually detected by rabbit polyclonal hyperimmune sera against an HPIV3 HN peptide (YWKHTNHGKDAGNELETC) and the recombinant purified ectodomain of the HPIV3 F protein, respectively. A mouse monoclonal GAPDH antibody (MilliporeSigma, Burlington, MA) was included to provide a loading control. Membranes were incubated with infrared dye-conjugated secondary goat anti-hamster (Rockland Immunochemicals, Limerick, PA), anti-rabbit and anti-mouse (LICORbio) antibodies. Images were acquired, the intensities of individual protein bands were quantified using Image Studio software (LICORbio), and normalized to GAPDH. Relative abundance of the B/HPIV3 proteins and HMPV F was presented as fold-change compared with those of the empty B/HPIV3 vector or the B/HPIV3 expressed HMPV wt F, respectively.

### Sucrose purification

LLC-MK2 cells were infected with recombinant viruses at an MOI of 0.1 PFU per cell and incubated at 32°C for 7 days in medium containing 2% FBS. HMPV was grown in the presence of 4% TrypLE Select (Life Technologies) in the medium without FBS. Viruses were purified by ultracentrifugation through 30%/60% discontinuous sucrose gradient and gently pelleted by centrifugation to remove sucrose. Virions were lysed in Pierce RIPA buffer (ThermoFisher Scientific), and the protein amount was quantified using Pierce Rapid Gold BCA Protein Assay Kit (ThermoFisher Scientific). One microgram of protein per virus was subjected to SDS-PAGE and Western blot as described above. Protein amounts were normalized to N and expressed as fold-change compared with B/HPIV3 control without insert in the same experiment. Amounts of HMPV F protein are shown relative to HMPV wt F expressed by B/HPIV3.

### Replication, immunogenicity, and protective efficacy in hamsters

Animal study protocols were approved by the National Institute of Allergy and Infectious Diseases Animal Care and Use Committee. To evaluate replication, 6-week-old golden Syrian hamsters in groups of 6, confirmed to be HPIV3 and HMPV seronegative by PRNT_60_, were inoculated IN with 10^5^ PFU of B/HPIV3 expressing different versions of HMPV F (Fig. 4A). B/HPIV3 without HMPV F insert and HMPV A were administered IN at a dose of 10^5^ and 10^6^ PFU, respectively, as controls. On day 3 p.i., hamsters were euthanized, and tissues, including nasal turbinates and lungs, were collected and homogenized. Virus in each clarified tissue homogenate was titrated on Vero cells by dual-antigen staining immunoplaque assay to determine the replication of viruses in the nasal turbinates and lungs as well as the stability of HMPV F insert expression in these tissues.

Immunogenicity was examined in another hamster study in which animals were immunized in the same manner and dosage as described above for the replication study (Fig. 5A). In addition, two groups (*n* = 6) were immunized with 20 µg of either of the two purified recombinant forms of HMPV pre-F protein (F-D185P or F-N46V/T160F), each mixed 1:1 (vol:vol) with alum-85 adjuvant (InvivoGen, San Diego, CA), and administered by IM injection at a final volume of 100 µL. Animals were boosted with an identical second dose 3 weeks later. Serum samples were collected from immunized hamsters at 4 weeks post-virus immunization or 2 weeks post-second IM injection. Two days after serum collection, all hamsters were challenged IN with 10^6.8^ PFU of HMPV (CAN97-83). At day 3 post-challenge, animals were euthanized, and nasal turbinates and lungs were collected for the quantification of HMPV replication. Tissue homogenates were prepared and titrated by HMPV plaque assay on Vero cells with data reported as PFU/g of each tissue.

### 60% plaque reduction neutralization assay (PRNT_60_)

The HPIV3-specific PRNT_60_ values of hamster sera were determined as previously described (50) on Vero cells using HPIV3 (JS strain) expressing green fluorescent protein. HMPV A- and B-neutralization assays were performed on Vero and LLC-MK2 cells, respectively. Briefly, sera were heated at 56°C for 30 min to inactivate serum complement and serial dilutions of each serum in duplicate were mixed with an equal volume of diluted virus (recombinant HMPV subgroup A, strain CAN97-83 (32) or HMPV B subgroup B2, strain CAN98-75 [54]). After incubation for 1 h at 37°C, the serum/virus mix was transferred onto cell monolayers and rocked for 3 h at 37°C. Vero and LLC-MK2 cells were washed and overlaid with Opti-MEM containing 0.8% methylcellulose and 4% or 2% TrypLE, respectively, and incubated for 7 days at 37°C. Plaque immunostaining was performed using a mixture of MPE8 and MPF5 antibodies as described above. Plaques were counted with ImageJ software (National Institutes of Health), and PRNT_60_ titers were calculated by linear regression analysis.

### Temperature sensitivity assay

Subconfluent monolayers of A549 or LLC-MK2 cells in 24-well plates were incubated with serial dilutions of the indicated viruses. After adsorption at 32°C and 5% CO_2_, 0.8% methylcellulose overlay in L-15 medium was added, and plates were incubated for 7 days (A549 cells) or 8 days (LLC-MK2 cells) at the indicated temperatures in closed stainless steel containers submerged in temperature-controlled water baths. The temperature sensitivity assay on A549 cells was repeated using methylcellulose overlay in F-12K medium, with a 7-day incubation in tissue culture incubators instead of water baths. Monolayers were fixed and immunostained to visualize plaques using a rabbit hyperimmune serum to PIV3 and human anti-HMPV F monoclonal antibodies MPE8 and MPF5, as described above.

## Data Availability

All data are included in the article.

## SUPPLEMENTAL MATERIAL

Supplemental material jvi.01145-25-s0001.pdf Figures S1 to S3 and Table S1.

ASM does not own the copyrights to Supplemental Material that may be linked to, or accessed through, an article. The authors have granted ASM a non-exclusive, world-wide license to publish the Supplemental Material files. Please contact the corresponding author directly for reuse.

## References

[1] Wang X, Li Y, Deloria-Knoll M, Madhi SA, Cohen C, Arguelles VL, Basnet S, Bassat Q, Brooks WA, Echavarria M, et al.. 2021. Global burden of acute lower respiratory infection associated with human parainfluenza virus in children younger than 5 years for 2018: a systematic review and meta-analysis. Lancet Glob Health 9:e1077–e1087. doi:10.1016/S2214-109X(21)00218-7 34166626 PMC8298256

[2] Glezen WP, Frank AL, Taber LH, Kasel JA. 1984. Parainfluenza virus type 3: seasonality and risk of infection and reinfection in young children. J Infect Dis 150:851–857. doi:10.1093/infdis/150.6.851 6094674

[3] Karron RA, Collins PL. 2013. Parainfluenza viruses, p 996–1023. In Knipe DM, Howley PM, Cohen JI, Griffin DE, Lamb RA, Martin MA, Racaniello VR, Roizman B (ed), Fields virology, 6th ed, Vol. 1. Lippincott Williams & Wilkins, Philadelphia.

[4] Moscona A. 2005. Entry of parainfluenza virus into cells as a target for interrupting childhood respiratory disease. J Clin Invest 115:1688–1698. doi:10.1172/JCI25669 16007245 PMC1159152

[5] Spriggs MK, Murphy BR, Prince GA, Olmsted RA, Collins PL. 1987. Expression of the F and HN glycoproteins of human parainfluenza virus type 3 by recombinant vaccinia viruses: contributions of the individual proteins to host immunity. J Virol 61:3416–3423. doi:10.1128/JVI.61.11.3416-3423.1987 2822951 PMC255937

[6] van Wyke Coelingh KL, Winter CC, Tierney EL, Hall SL, London WT, Kim HW, Chanock RM, Murphy BR. 1990. Antibody responses of humans and nonhuman primates to individual antigenic sites of the hemagglutinin-neuraminidase and fusion glycoproteins after primary infection or reinfection with parainfluenza type 3 virus. J Virol 64:3833–3843. doi:10.1128/JVI.64.8.3833-3843.1990 1695256 PMC249679

[7] van den Hoogen BG, de Jong JC, Groen J, Kuiken T, de Groot R, Fouchier RA, Osterhaus AD. 2001. A newly discovered human pneumovirus isolated from young children with respiratory tract disease. Nat Med 7:719–724. doi:10.1038/89098 11385510 PMC7095854

[8] Williams JV, Harris PA, Tollefson SJ, Halburnt-Rush LL, Pingsterhaus JM, Edwards KM, Wright PF, Crowe JE Jr. 2004. Human metapneumovirus and lower respiratory tract disease in otherwise healthy infants and children. N Engl J Med 350:443–450. doi:10.1056/NEJMoa025472 14749452 PMC1831873

[9] Wang X, Li Y, Deloria-Knoll M, Madhi SA, Cohen C, Ali A, Basnet S, Bassat Q, Brooks WA, Chittaganpitch M, et al.. 2021. Global burden of acute lower respiratory infection associated with human metapneumovirus in children under 5 years in 2018: a systematic review and modelling study. Lancet Glob Health 9:e33–e43. doi:10.1016/S2214-109X(20)30393-4 33248481 PMC7783516

[10] van den Hoogen BG, Herfst S, Sprong L, Cane PA, Forleo-Neto E, de Swart RL, Osterhaus A, Fouchier RAM. 2004. Antigenic and genetic variability of human metapneumoviruses. Emerg Infect Dis 10:658–666. doi:10.3201/eid1004.030393 15200856 PMC3323073

[11] Biacchesi S, Skiadopoulos MH, Boivin G, Hanson CT, Murphy BR, Collins PL, Buchholz UJ. 2003. Genetic diversity between human metapneumovirus subgroups. Virology (Auckl) 315:1–9. doi:10.1016/S0042-6822(03)00528-2 14592754

[12] Yang CF, Wang CK, Tollefson SJ, Piyaratna R, Lintao LD, Chu M, Liem A, Mark M, Spaete RR, Crowe JE, Williams JV. 2009. Genetic diversity and evolution of human metapneumovirus fusion protein over twenty years. Virol J 6:138. doi:10.1186/1743-422X-6-138 19740442 PMC2753315

[13] Papenburg J, Carbonneau J, Isabel S, Bergeron MG, Williams JV, De Serres G, Hamelin M-È, Boivin G. 2013. Genetic diversity and molecular evolution of the major human metapneumovirus surface glycoproteins over a decade. J Clin Virol 58:541–547. doi:10.1016/j.jcv.2013.08.029 24041471

[14] Skiadopoulos MH, Biacchesi S, Buchholz UJ, Riggs JM, Surman SR, Amaro-Carambot E, McAuliffe JM, Elkins WR, St Claire M, Collins PL, Murphy BR. 2004. The two major human metapneumovirus genetic lineages are highly related antigenically, and the fusion (F) protein is a major contributor to this antigenic relatedness. J Virol 78:6927–6937. doi:10.1128/JVI.78.13.6927-6937.2004 15194769 PMC421687

[15] Shirogane Y, Takeda M, Iwasaki M, Ishiguro N, Takeuchi H, Nakatsu Y, Tahara M, Kikuta H, Yanagi Y. 2008. Efficient multiplication of human metapneumovirus in Vero cells expressing the transmembrane serine protease TMPRSS2. J Virol 82:8942–8946. doi:10.1128/JVI.00676-08 18562527 PMC2519639

[16] Dutch RE, Jardetzky TS, Lamb RA. 2000. Virus membrane fusion proteins: biological machines that undergo a metamorphosis. Biosci Rep 20:597–612. doi:10.1023/a:1010467106305 11426696

[17] Ngwuta JO, Chen M, Modjarrad K, Joyce MG, Kanekiyo M, Kumar A, Yassine HM, Moin SM, Killikelly AM, Chuang G-Y, Druz A, Georgiev IS, Rundlet EJ, Sastry M, Stewart-Jones GBE, Yang Y, Zhang B, Nason MC, Capella C, Peeples ME, Ledgerwood JE, McLellan JS, Kwong PD, Graham BS. 2015. Prefusion F-specific antibodies determine the magnitude of RSV neutralizing activity in human sera. Sci Transl Med 7:309ra162. doi:10.1126/scitranslmed.aac4241 PMC4672383 26468324

[18] McLellan JS, Chen M, Leung S, Graepel KW, Du X, Yang Y, Zhou T, Baxa U, Yasuda E, Beaumont T, Kumar A, Modjarrad K, Zheng Z, Zhao M, Xia N, Kwong PD, Graham BS. 2013. Structure of RSV fusion glycoprotein trimer bound to a prefusion-specific neutralizing antibody. Science 340:1113–1117. doi:10.1126/science.1234914 23618766 PMC4459498

[19] Battles MB, Más V, Olmedillas E, Cano O, Vázquez M, Rodríguez L, Melero JA, McLellan JS. 2017. Structure and immunogenicity of pre-fusion-stabilized human metapneumovirus F glycoprotein. Nat Commun 8:1528. doi:10.1038/s41467-017-01708-9 29142300 PMC5688127

[20] Hsieh CL, Rush SA, Palomo C, Chou CW, Pickens W, Más V, McLellan JS. 2022. Structure-based design of prefusion-stabilized human metapneumovirus fusion proteins. Nat Commun 13:1299. doi:10.1038/s41467-022-28931-3 35288548 PMC8921277

[21] Lee YZ, Han J, Zhang YN, Ward G, Braz Gomes K, Auclair S, Stanfield RL, He L, Wilson IA, Zhu J. 2024. Rational design of uncleaved prefusion-closed trimer vaccines for human respiratory syncytial virus and metapneumovirus. Nat Commun 15:9939. doi:10.1038/s41467-024-54287-x 39550381 PMC11569192

[22] Ou L, Chen SJ, Teng I-T, Yang L, Zhang B, Zhou T, Biju A, Cheng C, Kong W-P, Morano NC, Stancofski E-S, Todd J-P, Tsybovsky Y, Wang S, Zheng C-Y, Mascola JR, Shapiro L, Woodward RA, Buchholz UJ, Kwong PD. 2023. Structure-based design of a single-chain triple-disulfide-stabilized fusion-glycoprotein trimer that elicits high-titer neutralizing responses against human metapneumovirus. PLoS Pathog 19:e1011584. doi:10.1371/journal.ppat.1011584 37738240 PMC10516418

[23] Kishko M, Stuebler A, Sasmal S, Chan Y, Huang D, Reyes C, Lin J, Price O, Kume A, Zong K, Bricault C, Alamares-Sapuay J, Zhang L. 2025. A computationally designed prefusion stabilized human metapneumovirus fusion protein vaccine antigen elicited a potent neutralization response. Vaccines (Basel) 13:523. doi:10.3390/vaccines13050523 40432132 PMC12115362

[24] Schmidt AC, McAuliffe JM, Murphy BR, Collins PL. 2001. Recombinant bovine/human parainfluenza virus type 3 (B/HPIV3) expressing the respiratory syncytial virus (RSV) G and F proteins can be used to achieve simultaneous mucosal immunization against RSV and HPIV3. J Virol 75:4594–4603. doi:10.1128/JVI.75.10.4594-4603.2001 11312329 PMC114212

[25] Karron RA, Thumar B, Schappell E, Surman S, Murphy BR, Collins PL, Schmidt AC. 2012. Evaluation of two chimeric bovine-human parainfluenza virus type 3 vaccines in infants and young children. Vaccine (Auckl) 30:3975–3981. doi:10.1016/j.vaccine.2011.12.022 PMC3509782 22178099

[26] Bernstein DI, Malkin E, Abughali N, Falloon J, Yi T, Dubovsky F, MI-CP149 Investigators. 2012. Phase 1 study of the safety and immunogenicity of a live, attenuated respiratory syncytial virus and parainfluenza virus type 3 vaccine in seronegative children. Pediatr Infect Dis J 31:109–114. doi:10.1097/INF.0b013e31823386f1 21926667

[27] Liang B, Ngwuta JO, Herbert R, Swerczek J, Dorward DW, Amaro-Carambot E, Mackow N, Kabatova B, Lingemann M, Surman S, Yang L, Chen M, Moin SM, Kumar A, McLellan JS, Kwong PD, Graham BS, Schaap-Nutt A, Collins PL, Munir S. 2016. Packaging and prefusion stabilization separately and additively increase the quantity and quality of respiratory syncytial virus (RSV)-neutralizing antibodies induced by an RSV fusion protein expressed by a parainfluenza virus vector. J Virol 90:10022–10038. doi:10.1128/JVI.01196-16 27581977 PMC5068507

[28] Biacchesi S, Pham QN, Skiadopoulos MH, Murphy BR, Collins PL, Buchholz UJ. 2006. Modification of the trypsin-dependent cleavage activation site of the human metapneumovirus fusion protein to be trypsin independent does not increase replication or spread in rodents or nonhuman primates. J Virol 80:5798–5806. doi:10.1128/JVI.00294-06 16731919 PMC1472577

[29] Liang B, Munir S, Amaro-Carambot E, Surman S, Mackow N, Yang L, Buchholz UJ, Collins PL, Schaap-Nutt A. 2014. Chimeric bovine/human parainfluenza virus type 3 expressing respiratory syncytial virus (RSV) F glycoprotein: effect of insert position on expression, replication, immunogenicity, stability, and protection against RSV infection. J Virol 88:4237–4250. doi:10.1128/JVI.03481-13 24478424 PMC3993740

[30] Liang B, Ngwuta JO, Surman S, Kabatova B, Liu X, Lingemann M, Liu X, Yang L, Herbert R, Swerczek J, Chen M, Moin SM, Kumar A, McLellan JS, Kwong PD, Graham BS, Collins PL, Munir S. 2017. Improved prefusion stability, optimized codon usage, and augmented virion packaging enhance the immunogenicity of respiratory syncytial virus fusion protein in a vectored-vaccine candidate. J Virol 91:e00189-17. doi:10.1128/JVI.00189-17 28539444 PMC5651718

[31] Liu X, Park HS, Matsuoka Y, Santos C, Yang L, Luongo C, Moore IN, Johnson RF, Garza NL, Zhang P, Lusso P, Best SM, Buchholz UJ, Le Nouën C. 2023. Live-attenuated pediatric parainfluenza vaccine expressing 6P-stabilized SARS-CoV-2 spike protein is protective against SARS-CoV-2 variants in hamsters. PLoS Pathog 19:e1011057. doi:10.1371/journal.ppat.1011057 37352333 PMC10325082

[32] Biacchesi S, Murphy BR, Collins PL, Buchholz UJ. 2007. Frequent frameshift and point mutations in the SH gene of human metapneumovirus passaged in vitro. J Virol 81:6057–6067. doi:10.1128/JVI.00128-07 17376897 PMC1900297

[33] Schmidt AC, McAuliffe JM, Huang A, Surman SR, Bailly JE, Elkins WR, Collins PL, Murphy BR, Skiadopoulos MH. 2000. Bovine parainfluenza virus type 3 (BPIV3) fusion and hemagglutinin-neuraminidase glycoproteins make an important contribution to the restricted replication of BPIV3 in primates. J Virol 74:8922–8929. doi:10.1128/jvi.74.19.8922-8929.2000 10982335 PMC102087

[34] Liu X, Luongo C, Matsuoka Y, Park HS, Santos C, Yang L, Moore IN, Afroz S, Johnson RF, Lafont BAP, Martens C, Best SM, Munster VJ, Hollý J, Yewdell JW, Le Nouën C, Munir S, Buchholz UJ. 2021. A single intranasal dose of a live-attenuated parainfluenza virus-vectored SARS-CoV-2 vaccine is protective in hamsters. Proc Natl Acad Sci USA 118. doi:10.1073/pnas.2109744118 PMC8685679 34876520

[35] Le Nouën C, Nelson CE, Liu X, Park H-S, Matsuoka Y, Luongo C, Santos C, Yang L, Herbert R, Castens A, Moore IN, Wilder-Kofie T, Moore R, Walker A, Zhang P, Lusso P, Johnson RF, Garza NL, Via LE, Munir S, Barber DL, Buchholz UJ. 2022. Intranasal pediatric parainfluenza virus-vectored SARS-CoV-2 vaccine is protective in monkeys. Cell 185:4811–4825. doi:10.1016/j.cell.2022.11.006 36423629 PMC9684001

[36] Tang RS, Schickli JH, MacPhail M, Fernandes F, Bicha L, Spaete J, Fouchier RAM, Osterhaus A, Spaete R, Haller AA. 2003. Effects of human metapneumovirus and respiratory syncytial virus antigen insertion in two 3’ proximal genome positions of bovine/human parainfluenza virus type 3 on virus replication and immunogenicity. J Virol 77:10819–10828. doi:10.1128/jvi.77.20.10819-10828.2003 14512532 PMC224993

[37] Tang RS, Mahmood K, MacPhail M, Guzzetta JM, Haller AA, Liu H, Kaur J, Lawlor HA, Stillman EA, Schickli JH, Fouchier RAM, Osterhaus ADME, Spaete RR. 2005. A host-range restricted parainfluenza virus type 3 (PIV3) expressing the human metapneumovirus (hMPV) fusion protein elicits protective immunity in African green monkeys. Vaccine (Auckl) 23:1657–1667. doi:10.1016/j.vaccine.2004.10.009 PMC7115684 15705469

[38] Groen K, van Nieuwkoop S, Meijer A, van der Veer B, van Kampen JJA, Fraaij PL, Fouchier RAM, van den Hoogen BG. 2023. Emergence and potential extinction of genetic lineages of human metapneumovirus between 2005 and 2021. mBio 14:e0228022. doi:10.1128/mbio.02280-22 36507832 PMC9973309

[39] Muñoz-Escalante JC, Mata-Moreno G, Rivera-Alfaro G, Noyola DE. 2022. Global extension and predominance of human metapneumovirus A2 genotype with partial G gene duplication. Viruses 14:1058. doi:10.3390/v14051058 35632799 PMC9146545

[40] McLellan JS, Chen M, Joyce MG, Sastry M, Stewart-Jones GBE, Yang Y, Zhang B, Chen L, Srivatsan S, Zheng A, et al.. 2013. Structure-based design of a fusion glycoprotein vaccine for respiratory syncytial virus. Science 342:592–598. doi:10.1126/science.1243283 24179220 PMC4461862

[41] Venkatesan P. 2023. First RSV vaccine approvals. Lancet Microbe 4:e577. doi:10.1016/S2666-5247(23)00195-7 37390835

[42] Ottolini MG, Porter DD, Hemming VG, Prince GA. 2000. Enhanced pulmonary pathology in cotton rats upon challenge after immunization with inactivated parainfluenza virus 3 vaccines. Viral Immunol 13:231–236. doi:10.1089/vim.2000.13.231 10893002

[43] Belshe RB, Edwards KM, Vesikari T, Black SV, Walker RE, Hultquist M, Kemble G, Connor EM, CAIV-T Comparative Efficacy Study Group. 2007. Live attenuated versus inactivated influenza vaccine in infants and young children. N Engl J Med 356:685–696. doi:10.1056/NEJMoa065368 17301299

[44] Vesikari T. 2008. Emerging data on the safety and efficacy of influenza vaccines in children. Pediatr Infect Dis J 27:S159–S161. doi:10.1097/INF.0b013e31818a545d 18955892

[45] Rush SA, Brar G, Hsieh CL, Chautard E, Rainho-Tomko JN, Slade CD, Bricault CA, Kume A, Kearns J, Groppo R, Mundle ST, Zhang L, Casimiro D, Fu TM, DiNapoli JM, McLellan JS. 2022. Characterization of prefusion-F-specific antibodies elicited by natural infection with human metapneumovirus. Cell Rep 40:111399. doi:10.1016/j.celrep.2022.111399 36130517

[46] Wellford SA, Moseman AP, Dao K, Wright KE, Chen A, Plevin JE, Liao TC, Mehta N, Moseman EA. 2022. Mucosal plasma cells are required to protect the upper airway and brain from infection. Immunity 55:2118–2134. doi:10.1016/j.immuni.2022.08.017 36137543 PMC9649878

[47] Liang B, Matsuoka Y, Le Nouën C, Liu X, Herbert R, Swerczek J, Santos C, Paneru M, Collins PL, Buchholz UJ, Munir S. 2020. A parainfluenza virus vector expressing the respiratory syncytial virus (RSV) prefusion F protein is more effective than RSV for boosting a primary immunization with RSV. J Virol 95:e01512-20. doi:10.1128/JVI.01512-20 33115876 PMC7944453

[48] Buchholz UJ, Finke S, Conzelmann KK. 1999. Generation of bovine respiratory syncytial virus (BRSV) from cDNA: BRSV NS2 is not essential for virus replication in tissue culture, and the human RSV leader region acts as a functional BRSV genome promoter. J Virol 73:251–259. doi:10.1128/JVI.73.1.251-259.1999 9847328 PMC103829

[49] Durbin AP, Hall SL, Siew JW, Whitehead SS, Collins PL, Murphy BR. 1997. Recovery of infectious human parainfluenza virus type 3 from cDNA. Virology (Auckl) 235:323–332. doi:10.1006/viro.1997.8697 9281512

[50] Liu X, Liang B, Liu X, Amaro-Carambot E, Surman S, Kwong PD, Graham BS, Collins PL, Munir S. 2020. Human parainfluenza virus type 3 expressing the respiratory syncytial virus pre-fusion F protein modified for virion packaging yields protective intranasal vaccine candidates. PLoS One 15:e0228572. doi:10.1371/journal.pone.0228572 32045432 PMC7012412

[51] Corti D, Bianchi S, Vanzetta F, Minola A, Perez L, Agatic G, Guarino B, Silacci C, Marcandalli J, Marsland BJ, Piralla A, Percivalle E, Sallusto F, Baldanti F, Lanzavecchia A. 2013. Cross-neutralization of four paramyxoviruses by a human monoclonal antibody. Nature 501:439–443. doi:10.1038/nature12442 23955151

[52] Stewart-Jones GBE, Gorman J, Ou L, Zhang B, Joyce MG, Yang L, Cheng C, Chuang G-Y, Foulds KE, Kong W-P, Olia AS, Sastry M, Shen C-H, Todd J-P, Tsybovsky Y, Verardi R, Yang Y, Collins PL, Corti D, Lanzavecchia A, Scorpio DG, Mascola JR, Buchholz UJ, Kwong PD. 2021. Interprotomer disulfide-stabilized variants of the human metapneumovirus fusion glycoprotein induce high titer-neutralizing responses. Proc Natl Acad Sci USA 118:e2106196118. doi:10.1073/pnas.2106196118 34551978 PMC8488613

[53] Ulbrandt ND, Ji H, Patel NK, Riggs JM, Brewah YA, Ready S, Donacki NE, Folliot K, Barnes AS, Senthil K, Wilson S, Chen M, Clarke L, MacPhail M, Li J, Woods RM, Coelingh K, Reed JL, McCarthy MP, Pfarr DS, Osterhaus A, Fouchier RAM, Kiener PA, Suzich JA. 2006. Isolation and characterization of monoclonal antibodies which neutralize human metapneumovirus in vitro and in vivo. J Virol 80:7799–7806. doi:10.1128/JVI.00318-06 16873237 PMC1563801

[54] Peret TCT, Boivin G, Li Y, Couillard M, Humphrey C, Osterhaus A, Erdman DD, Anderson LJ. 2002. Characterization of human metapneumoviruses isolated from patients in North America. J Infect Dis 185:1660–1663. doi:10.1086/340518 12023774 PMC7109943

