# Supplementary material for: Intranasal bovine/human parainfluenza virus 3 vaccine candidates expressing human metapneumovirus wild-type or pre-fusion F protein elicit protective immunity against human metapneumovirus in hamsters
Source: J Virol. 2025 Nov 25;99(12):e01145-25. doi: 10.1128/jvi.01145-25 (PMC12724368; doi:10.1128/jvi.01145-25)
Supplement: Supplemental material — Figures S1 to S3 and Table S1. [file jvi.01145-25-s0001.pdf]

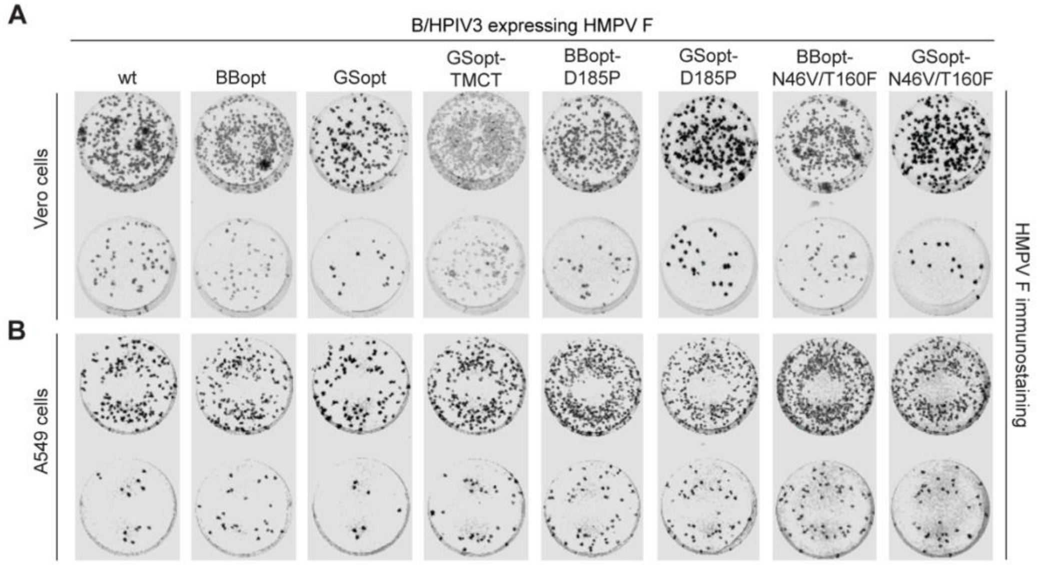

Supplemental figure 1. HMPV F expression by B/HPIV3 (related to figure 1). Data from figure 1 C and D are shown here in A and B, respectively, depicting only HMPV F staining. **A, B** Vero and A549 cells in 24-well plates were infected with serially diluted P2 virus stocks, covered with a methylcellulose overlay and incubated for 7 days. Cells were fixed and plaques were immunostained for the PIV3 and HMPV F antigens detected with infrared dye conjugated antibodies. Staining for HMPV F in Vero (**A**) and A549 (**B**) cells is shown in shades of black.

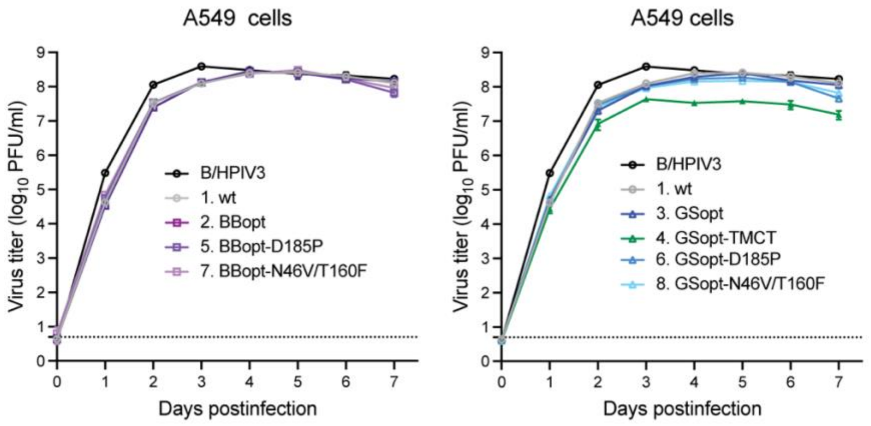

Supplemental figure 2. Replication kinetics of recombinant viruses in A549 cells (related to figure 2; the experiment as described in Fig. 2 was performed here, to examine multicycle growth of B/HPIV3 vectors, with infection starting at a lower MOI). A549 cells in six-well plates were infected in triplicate with indicated viruses at an MOI of 0.01 PFU per cell and incubated at 32°C for a total of 7 days. At 24 h intervals, aliquots of culture medium were collected and replaced with equal volume of fresh medium. Virus titers were determined by immunoplaque assay on Vero cells. The mean titers (±SD) are shown as two separate graph panels for visual clarity.

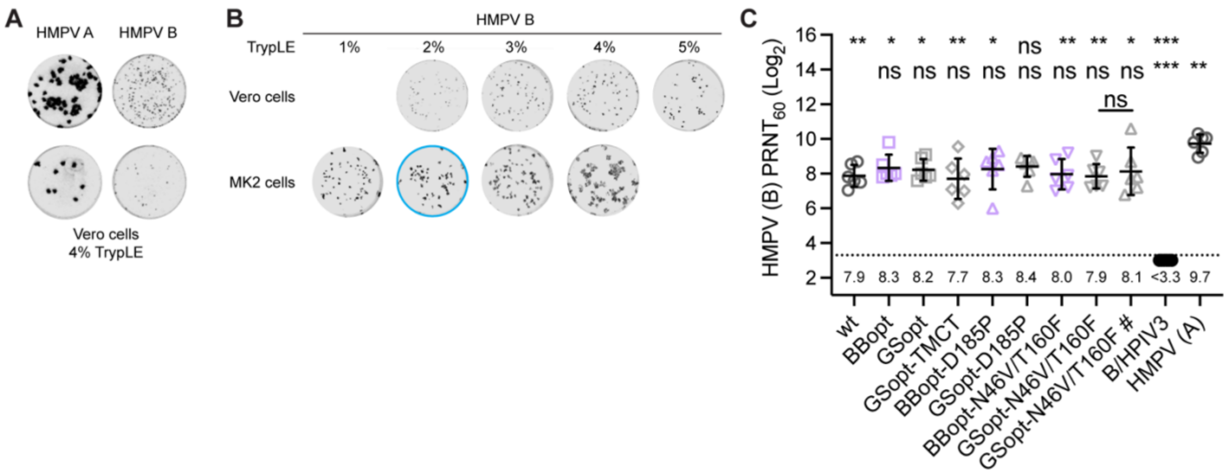

Supplemental figure 3. Hamster serum-HMPV B neutralizing antibody titers (related to figure 5). **A,** Comparison of HMPV A and HMPV B plaque formation on Vero cells under an overlay containing 0.8% methylcellulose and 4% TrypLE. Plaques are shown from two wells infected with tenfold dilutions of HMPV A or HMPV B. **B,** HMPV B plaque formation on Vero and LLC-MK2 cells under overlays containing different percentages of TrypLE. The well outlined in blue shows the optimal conditions chosen for determining the HMPV B $PRNT_{60}$ of the hamster sera. **C,** Sera from hamsters immunized with the candidate vaccine viruses were analyzed for the neutralizing antibodies against HMPV B by $PRNT_{60}$ on LLC-MK2 cells. * $p<0.05$, ** $p<0.01$, *** $p<0.001$, ns – not significant ($p>0.05$) by one-way ANOVA and Dunnett's multiple-comparisons test.

Supplemental table 1. Temperature sensitivity and plaque phenotype of B/HPIV3/GSopt-N46V/T160F # in LLC-MK2 cells

	Virus titer (log_{10} PFU/ml) at temperature (°C)[a]							Lowest restrictive temperature		
Virus[b]	32	35	36	37	38	39	40	T_{SP}[c]	T_{MP}[d]	T_{SH}[e]
rHPIV3 JS	8.5	8.6	8.5	8.4	8.5	8.5	8.4	>40	>40	>40
rHPIV3 JS wtHN	8.7	8.7	8.7	8.7	8.6	8.6	8.6	>40	>40	>40
B/HPIV3	6.4	6.4	6.5	6.4	6.5	6.4 [c]	6.0 [d]	39	40	>40
B/HPIV3-wtHN	7.6	7.6	7.5	7.4	7.4	7.5 [c]	7.0 [d]	39	40	>40
B/HPIV3/GSopt/N46V/T160F#	6.9	6.8	6.9	6.8	6.9 [c]	6.7 [c]	5.7 [d]	38	40	>40

[a] *The temperature sensitivity phenotype for each virus was evaluated by plaque assay in LLC-MK2 cells at the indicated temperatures.*

[b] *B/HPIV3 represents a reference control of a vaccine candidate that was attenuated in HPIV3 seronegative infants and children (28, 35). rHPIV3 JS and rHPIV JS wtHN, as well as B/HPIV3 and B/HPIV3-wtHN, differ only at the wtHN amino acid assignments 263 and 370 (Materials and Methods).*

[c] *T_{SP} (small-plaque temperature) is defined as the lowest restrictive temperature at which the small-plaque phenotype is observed.*

[d] *T_{MP} (micro-plaque temperature) is defined as the lowest restrictive temperature at which the micro-plaque phenotype is observed.*

[e] *T_{SH}, Shutoff temperature; defined as the lowest restrictive temperature at which the reduction compared to 32°C is 100-fold or greater than that observed for the wild-type virus at the two temperatures.*
